# Determination of Microbial Maintenance in Acetogenesis and Methanogenesis by Experimental and Modeling Techniques

**DOI:** 10.3389/fmicb.2019.00166

**Published:** 2019-02-08

**Authors:** Fabian Bonk, Denny Popp, Sören Weinrich, Heike Sträuber, Daniela Becker, Sabine Kleinsteuber, Hauke Harms, Florian Centler

**Affiliations:** ^1^Department of Environmental Microbiology, UFZ-Helmholtz Centre for Environmental Research, Leipzig, Germany; ^2^Biochemical Conversion Department, DBFZ-Deutsches Biomasseforschungszentrum gGmbH, Leipzig, Germany

**Keywords:** decay rate, non-growth associated maintenance, maintenance coefficient, apparent yield, dilution rate, hydraulic retention time, chemostat, Anaerobic Digestion Model No. 1

## Abstract

For biogas-producing continuous stirred tank reactors, an increase in dilution rate increases the methane production rate as long as substrate input can be converted fully. However, higher dilution rates necessitate higher specific microbial growth rates, which are assumed to have a strong impact on the apparent microbial biomass yield due to cellular maintenance. To test this, we operated two reactors at 37°C in parallel at dilution rates of 0.18 and 0.07 days^-1^ (hydraulic retention times of 5.5 and 14 days, doubling times of 3.9 and 9.9 days in steady state) with identical inoculum and a mixture of volatile fatty acids as sole carbon sources. We evaluated the performance of the Anaerobic Digestion Model No. 1 (ADM1), a thermodynamic black box approach (TBA), and dynamic flux balance analysis (dFBA), to describe the experimental observations. All models overestimated the impact of dilution rate on the apparent microbial biomass yield when using default parameter values. Based on our analysis, a maintenance coefficient value below 0.2 kJ per carbon mole of microbial biomass per hour should be used for the TBA, corresponding to 0.12 mmol ATP per gram dry weight per hour for dFBA, which strongly deviates from the value of 9.8 kJ Cmol h^-1^ that has been suggested to apply to all anaerobic microorganisms at 37°C. We hypothesized that a decrease in dilution rate might select taxa with minimized maintenance expenditure. However, no major differences in the dominating taxa between the reactors were observed based on amplicon sequencing of 16S rRNA genes and terminal restriction fragment length polymorphism analysis of *mcrA* genes. Surprisingly, *Methanosaeta* dominated over *Methanosarcina* even at a dilution rate of 0.18 days^-1^, which contradicts previous model expectations. Furthermore, only 23–49% of the bacterial reads could be assigned to known syntrophic fatty acid oxidizers, indicating that unknown members of this functional group remain to be discovered. In conclusion, microbial maintenance was found to be much lower for acetogenesis and methanogenesis than previously assumed, likely due to the exceptionally low growth rates in anaerobic digestion. This finding might also be relevant for other microbial systems operating at similarly low growth rates.

## Introduction

Anaerobic digestion (AD) is a key technology in environmental biotechnology and renewable energy supply (Plugge, 2017). In AD, microorganisms convert complex organic materials such as organic waste to biogas (Plugge, 2017). Mechanistic models have been shown to be useful for the simulation and control of the biogas process (Lauwers et al., 2013). It has been suggested that the further inclusion of microbial community data might lead to drastic improvements in model performance (Lauwers et al., 2013). In this study, we analyzed the effect of dilution rate on the microbial community with a focus on the influence of dilution rate and microbial maintenance on microbial biomass concentration.

The dilution rate is a crucial process parameter for continuous stirred tank reactors (CSTR), which is one of the most prevalent reactor configurations for AD around the world, and particularly in Germany (Weiland, 2010). The dilution rate is the reciprocal of the hydraulic retention time (HRT). To increase the organic loading rate (OLR) of a CSTR, with the aim of higher productivity, the substrate concentration in the feed and/or the dilution rate can be increased. The possibilities to increase the substrate concentration in the influent are limited, however, for example due to pumping or mixing problems at high solid contents. Therefore, increasing the dilution rate is the easiest way to reach higher OLRs. However, increasing the dilution rate can lead to process instability and even process failure (Ziganshin et al., 2016). In a CSTR, there is no biomass retention or recycling and therefore, the higher the dilution rate, the faster microbial biomass is washed out of the CSTR, and hence, higher specific microbial growth rates are required to compensate for this loss. Therefore, understanding the impact of dilution rate on the microbial community is crucial for process optimization. Among other effects, the dilution rate can affect microbial biomass concentration (Heijnen, 2002) and microbial community composition (Ziganshin et al., 2016).

The impact of the dilution rate on the microbial biomass concentration is important because the capacity of a digester to convert substrate depends on the amount of available biocatalysts (Van Lier et al., 2015), i.e., the amount of microbial biomass in the digester. Both negative and positive relationships between specific microbial growth rate and apparent microbial biomass yield (and thereby biomass concentration) have been found in the broader field of microbial ecology (Lipson, 2015). However, these relationships have received little attention in AD research.

Positive relationships between specific microbial growth rate and apparent microbial biomass yield were found in various experiments and hypothesized to be a result of substrate usage for processes other than growth, such as cellular maintenance (Lipson, 2015). At low specific microbial growth rates, energy usage for these non-growth related processes compared to growth-related processes becomes larger, resulting in a smaller apparent microbial biomass yield (*Y_app_*). Apparent microbial biomass yield refers to the net microbial biomass increase per substrate consumed. The maximum microbial biomass yield (*Y_max_*), in contrast, is a theoretical construct that describes the gross microbial biomass increase per substrate consumed if non-growth related processes are neglected. Non-growth related processes include (a) osmoregulation, (b) defense against oxidative stress, (c) cell motility, (d) synthesis, repair and turnover of macromolecules, (e) energy spilling reactions, (f) shifts in metabolic pathways, (g) changes in storage of polymeric carbon compounds and (h) extracellular losses of compounds not involved in osmoregulation (Van Bodegom, 2007). The processes (a–d) were termed “physiological maintenance” (Van Bodegom, 2007). Kempes et al. (2017) further differentiated between “maintenance metabolism” (inferred as substrate use under zero-growth conditions), “endogenous metabolism” (substrate use of cells after starvation), and “basal power requirement” (true minimum substrate use of a cell).

This diversity of non-growth related processes has led to the formulation of conceptually sound but parameter-rich models (Kempes et al., 2017; Van Bodegom, 2007). Van Bodegom (2007) acknowledged the lack of experimental data to determine all individual non-growth variables in such models. This makes them difficult to apply to complex microbial communities such as AD communities because these parameters would need to be determined for hundreds of species. In AD models, microbial maintenance is usually considered following one of two basic concepts: the “Herbert Model” and the “Pirt Model” (Wang and Post, 2012). In the “Herbert Model,” microbial maintenance is implemented as a negative specific growth rate. This concept is used, for example, in the Anaerobic Digestion Model No.1 (ADM1). In the “Pirt Model,” microbial maintenance is expressed in the form of an energy consumption rate. This approach is applied as a maintenance coefficient in the thermodynamic black box approach (TBA) and as non-growth associated maintenance (NGAM) in flux balance analysis (FBA).

Besides the positive relationship between specific microbial growth rate and apparent yield, Lipson (2015) pointed out that there is additionally both theoretical and empirical support for a negative relationship. For example, costly additional protein synthesis might be required to achieve higher specific microbial growth rates (Wong et al., 2009). Both linear and sigmoidal negative relationships have been suggested (Lipson, 2015).

**FIGURE 1:**
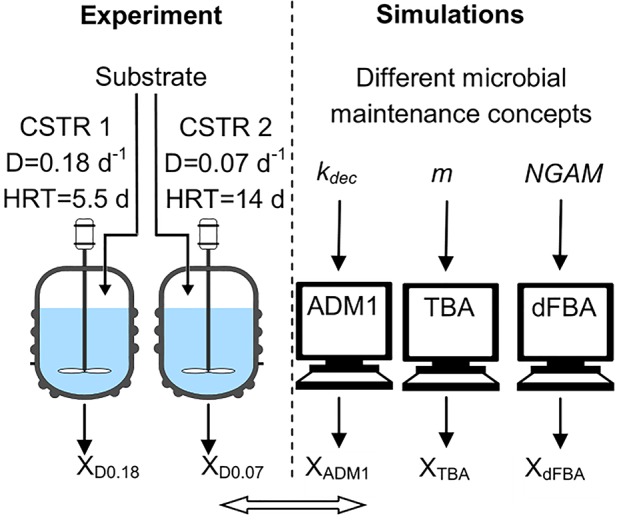
Study design. Substrate: VFAs and mineral media; ADM1: Anaerobic Digestion Model No.1; CSTR: continuous stirred tank reactor; D: dilution rate; dFBA: dynamic flux-balance analysis; HRT: hydraulic retention time; *k_dec_*: decay rate; *m*: maintenance coefficient; *NGAM*: non-growth associated maintenance; TBA: thermodynamic black box approach; VFA: volatile fatty acid; X: microbial biomass concentration, X_D0.18_: X of CSTR with D = 0.18 d^-1^.

The aim of this study was to analyze the impact of dilution rate and microbial maintenance on the microbial biomass concentration in acetogenesis and methanogenesis (Figure 1). Two laboratory scale CSTRs were fed with synthetic media containing a mixture of volatile fatty acids (VFA) as the sole carbon source, both started with an identical inoculum and operated in parallel but at different dilution rates (0.18 and 0.07 days^-1^). Final microbial biomass concentrations were compared to predictions from the three models applied in AD research: ADM1 (Herbert Model), TBA (Pirt Model) and a dynamic FBA model (dFBA, Pirt Model). These models all account for microbial maintenance but implemented following different concepts and with different default values, and therefore are expected to lead to different predictions concerning the impact of dilution rate on microbial biomass concentrations. In addition, the composition of the microbial communities was analyzed using 16S rRNA gene amplicon sequencing targeting bacteria and terminal restriction fragment length polymorphism (T-RFLP) analysis of the *mcrA* gene targeting methanogenic archaea.

## Materials and Methods

### Laboratory-Scale CSTR Experiments

Two laboratory-scale CSTRs with 6 L working volume were operated in parallel over 70 days at 37°C but with different dilution rates (0.18 and 0.07 days^-1^). Both CSTRs were fed continuously with a synthetic, liquid substrate containing a mixture of VFAs as the only carbon source (45% acetic, 45% butyric and 10% propionic acid based on chemical oxygen demand, COD) with a total concentration of 37.2 gCOD L^-1^ in a mineral medium containing all necessary trace elements, macronutrients and vitamins (see Supplementary Table S1, Supplementary Material A). The CSTRs were inoculated from a lab-scale digester operated at a dilution rate of 0.125 days^-1^ with the same synthetic substrate for 7 months. Biogas production rate, biogas composition (CH_4_, CO_2_, O_2_, H_2_, and H_2_S), VFA concentrations, total solids content (TS), volatile solids content (VS), and pH were determined as described previously (Mulat et al., 2016). The TS and VS content measurements were modified in a way that a reactor sample was not added directly to a crucible, but only the pellet after centrifuging 100 mL reactor content (10,000 × g, 10 min, and 10°C) to increase the amount of biomass per analysis and to reduce the potential error of VFAs in the sample.

### Microbial Community Analyses

#### DNA Extraction

Samples (1.5 mL) from the lab-scale CSTRs were centrifuged at -7°C and 15,000 × g for 2 min and the supernatant was discarded. The samples did not freeze within the 2 min despite the low temperature. The pellets were stored at -20°C until DNA extraction. DNA was extracted from the pellets using the NucleoSpin^®^Soil Kit (MACHEREY-NAGEL GmbH & Co., KG, Germany, buffer SL2, no enhancer). Purified DNA quantity and quality were determined by agarose gel electrophoresis and by NanoDrop^®^ND 1000 spectral photometer (Thermo Fisher Scientific, United States) and the DNA was stored at -20°C.

#### Composition of Methanogenic Archaea

The methanogenic community composition was assessed by T-RFLP analysis of *mcrA* gene amplicons as described previously (Sträuber et al., 2016). For the taxonomic assignment, the database of Bühligen et al. (2016) was used. Primers used were mlas (GGTGGTGTMGGDTTCACMCARTA) and mcrA-rev (CGTTCATBGCGTAGTTVGGRTAGT) and PCR conditions were applied as described previously (Steinberg and Regan, 2008). Restriction enzymes used were *Mwo*I and *Bst*NI (New England Biolabs). Methanogens contain only one copy of the *mcrA* gene per genome (Steinberg and Regan, 2008). However, Methanobacteriales and Methanococcales additionally contain one copy of the *mrtA* gene, which is also amplified by the primers mlas and mcrA-rev (Steinberg and Regan, 2008). Therefore, relative T-RF abundances of these orders were corrected by a factor of 2.

#### Composition of the Bacterial Communities

Bacterial community compositions of both CSTRs at the beginning and at the end of the experiment were analyzed by amplicon sequencing of 16S rRNA genes. PCR amplification and amplicon sequencing using the MiSeq platform (V3, 2x300bp, Illumina) were performed by LGC Genomics GmbH (Berlin, Germany). The V3-V4 regions of the 16S rRNA genes were amplified using the primers 341f (CCTACGGGNGGCWGCAG) and 785r (GACTACHVGGGTATCTAAKCC) according to Klindworth et al. (2013). Initial bioinformatics preparation of paired-end reads was performed by LGC Genomics including de-multiplexing, removal of barcodes (allowing 1 mismatch), adapter and primer sequences (allowing 3 mismatches), and merging of forward and reverse reads using the BBMerge 34.48 software^1^. Merged reads were further processed with the QIIME 1.9.1 Virtual Box release (Caporaso et al., 2010). Reads were quality-filtered removing low quality reads (quality threshold lower than 20) and allowing no ambiguous base calls. Removal of chimeric sequences and clustering into operational taxonomic units (OTUs) was achieved by the usearch tool (Edgar, 2010). For taxonomic assignment, the latest MiDAS taxonomy 2.1 (McIlroy et al., 2015) and the RDP Classifier 2.2 (confidence threshold 0.8) were used (Wang et al., 2007). For downstream analyses, the OTU table was rarefied to 23,074 sequences per sample. Rarefaction curves can be found in the (Supplementary Figure S6, Supplementary Material A). In the resulting OTU table, only bacterial OTUs were retained as the applied primers only partially amplify archaeal 16S rRNA genes and would result in a strongly biased methanogenic community composition (Klindworth et al., 2013). Raw de-multiplexed sequence data has been deposited at EMBL European Nucleotide Archive (ENA) under accession number PRJEB22603^2^.

The relative abundances of 16S rRNA genes were corrected by the average 16S rRNA gene copy number per genome. The 16S rRNA gene copy numbers per genome for each OTU at genus level were taken from the rrnDB database^3^ version 5.2 using the taxonomy search function (Name type: NCBI – all names, accessed on September 5, 2017). If no copy number was available at the genus level, the next higher taxonomic level was chosen.

### Simulations

#### ADM1 (Herbert Model)

The Herbert Model describes endogenous metabolism by a negative specific microbial growth rate (Wang and Post, 2012) and, therefore, technically has the form of a biomass decay term:

(1) $$\mu \;(S)=\mu_{max}\cdot \frac{S}{S+K_{S}}-a,$$

with μ being the substrate concentration dependent specific microbial growth rate, μ_*max*_ the maximum specific microbial growth rate, *S* the substrate concentration, *K_S_* the half saturation constant and *a* the specific maintenance rate. This model for maintenance in the form of biomass decay is used in the ADM1 as the constant parameter *k_dec_* (equals *a* in Equation 1).

A COD-based ADM1 implementation (Rosen and Jeppsson, 2006) was used in this study, which includes minor changes to the original model structure of Batstone et al. (2002), such as additional balancing terms for inorganic nitrogen and carbon to ensure closed nitrogen and carbon balances in all 19 processes, the utilization of Hill functions to calculate pH inhibition and the calculation of the biogas production rate based on an overpressure in the headspace. All simulations were performed until steady state was reached. Thus, the influence of initial concentrations for individual state variables can be neglected.

Default parameters as introduced by Rosen and Jeppsson (2006) were used. The most important of these default parameters for our study were *Y_ac_* (0.05 gCOD_X_ gCOD_S_^-1^), *Y_pro_* (0.04), *Y_*c4*_* (0.06) and *Y_*h2*_* (0.06), which are the microbial biomass yields of acetoclastic methanogens, propionic acid oxidizers, butyric acid oxidizers and hydrogenotrophic methanogens, respectively, and the microbial decay rate *k_dec_* (0.02 days^-1^).

In the original structure of ADM1, decaying microbial biomass is assumed to disintegrate into carbohydrates, lipids and proteins, which are hydrolyzed and used by bacterial populations as substrate. In this way, some of the biomass lost due to maintenance can be utilized for microbial growth, which is not the case in the Herbert Model. However, this recycling of dead biomass is negligible, as for the default parameters and the dilution rate of 0.07 days^-1^, the biomass recycling of acetogens and methanogens only accounts for 0.54% of the respective population biomass (see Supplementary Table S3). Therefore, we interpret ADM1’s *k_dec_* as maintenance coefficient of the Herbert Model.

For better comparison with the TBA (Equation 5b), Equation 2 for the apparent yield *Y_app_* was derived for steady state (see Supplementary Material A, section “Derivation of Equation 2”) based on the dilution rate *D*, the decay rate *k_dec_* and the microbial biomass yield *Y*:

(2) $$Y_{app}=\frac{Y}{\frac{k_{dec}}{D}+1},$$

#### TBA (Pirt Model)

In the Pirt Model, substrate consumption is divided into substrate for growth and substrate to fulfill maintenance requirements:

(3) $$q(S)=\frac{\mu_{max}\cdot \frac{S}{S+K_{S}}}{Y_{max}}+m,$$

with *q* being the specific substrate uptake rate, μ_*max*_ the maximum specific microbial growth rate, *S* the substrate concentration, *K* the half saturation constant, *Y_max_* the maximum yield and *m* the maintenance coefficient. In AD research, it has been applied by Wandrey and Aivasidis (1983). The maintenance coefficient *m* has been implemented by Heijnen (2002) in a TBA model as a universal energy requirement, which is mainly influenced by the temperature and only little influenced by the type of microorganism, electron donor, and electron acceptor. This universal energy requirement is particularly useful for complex microbial communities because no parameters need to be fitted.

For steady state, the biomass concentration in the CSTRs for the TBA can be calculated based on the apparent yield *Y_app_*, the substrate influent concentration *S_in_*, the substrate production rate from other reactions (e.g., acetic acid production from butyric acid oxidation) ${\dot{S}}_{i}$, the dilution rate *D*, and the substrate concentration *S* inside the CSTR following Equation 4.

(4) $$X=Y_{app}\cdot \left(S_{in}+\frac{{\dot{S}}_{i}}{D}-S\right),$$

Heijnen (2002) described a TBA for microbial growth with an apparent yield *Y_app_*, which is derived from the maximum yield *Y_max_* diminished by a maintenance coefficient *m* and the specific microbial growth rate μ (Equation 5a), which can be rewritten to Equation 5b assuming a CSTR in steady state.

(5a) $$Y_{app}=\frac{Y_{max}}{1+\frac{m.Y_{max}}{\mu }},$$

(5b) $$Y_{app}=\frac{Y_{max}}{1+\frac{m.Y_{max}}{D}},$$

Maintenance coefficients *m* for each substrate were calculated based on the Gibbs energy of 9.8 kJ per carbon mole microbial biomass (Cmol_X_) per hour at 37°C based on Tijhuis et al. (1993). The maintenance coefficient was converted to substrate consumption rates by the Gibbs energy change of catabolism as presented by Kleerebezem and Van Loosdrecht (2010). *Y_max_* values were based on the Gibbs energy dissipation method (Kleerebezem and Van Loosdrecht, 2010, see Supplementary Table S7). Carbon mole based units were converted to mass-base assuming a microbial biomass composition of CH_1.8_O_0.5_N_0.2_ (Kleerebezem and Van Loosdrecht, 2010). See section “Details on the Approach Used in this Study” in Supplementary Material A for detailed explanations.

#### dFBA

FBA modeling relies on metabolic stoichiometric networks, which detail the intracellular conversion of metabolites. Assuming that intracellular metabolites are at steady state and that the cell maximizes its growth, this approach allows for the prediction of specific microbial growth rate, intracellular metabolic fluxes and product synthesis rates, given a substrate uptake rate (Orth et al., 2010). By restricting the steady state assumption to short time intervals, dFBA allows for the dynamic simulation of microbial populations (Mahadevan et al., 2002). We simulated a chemostat containing a monoclonal population with biomass concentration *X* (g L^-1^) being fed with a growth limiting substrate *S* (g L^-1^, either being acetic, propionic, butyric acid or hydrogen) according to:

(6) $$\frac{dX}{dt}=\mu \cdot X-D\cdot X$$

(7) $$\frac{dS}{dt}=(S_{in}-S)\cdot D-v_{S}(S)\cdot X$$

with dilution rate *D* (d^-1^) and substrate concentration in the inflow *S_in_* (g L^-1^). Following the dFBA approach, the specific microbial growth rate of the population μ (d^-1^) and the specific substrate uptake *v_s_* (g gDW^-1^ d^-1^) were computed by optimizing intracellular flux distributions within metabolic networks with respect to cellular growth. The current maximal specific substrate uptake rate *v_uptake_* (g gDW^-1^ d^-1^) was computed based on current substrate concentration *S* in the reactor according to *v_uptake_* = *v_max_ S/(K_S_ + S)* with maximal specific uptake rate *v_max_* (g gDW^-1^ d^-1^) and half saturation constant *K_S_* (g L^-1^). To numerically solve the model, an Euler method was implemented in Matlab R2015b (The MathWorks, Inc., Natick, MA, United States), assuming linear dynamics between iteration steps, and using CellNetAnalyzer for FBA computations (Klamt et al., 2007). Simulations for the two dilution rates of 0.18 and 0.07 days^-1^ were run until steady state was reached, using a time step size of 1.5 × 10^-6^ days. For acetoclastic and hydrogenotrophic growth, the *Methanosarcina barkeri* model iMG746 (Gonnerman et al., 2013) was used, in which NGAM requirement is fixed to 2 mmol ATP gDW^-1^ h^-1^ (Gonnerman et al., 2013). In separate simulations, either acetoclastic (choosing acetic acid as the growth limiting substrate with *S_in_* = 29.76 g L^-1^, *v_max_* = 9.78 g gDW^-1^ d^-1^, *K_S_* = 0.2952 g L^-1^) or hydrogenotrophic methanogenesis of *M. barkeri* was simulated (choosing hydrogen as the growth limiting substrate with *S_in_* = 622.7 mg L^-1^, *v_max_* = 2.5885 g gDW^-1^ d^-1^, *K_S_* = 26.208 μg L^-1^). *S_in_* values were calculated as acetic acid or hydrogen equivalents based on the experimental mixed feed composition. *K_S_* values were taken from Oude Elferink et al. (1994) and *v_max_* values adjusted so that model-predicted maximal specific microbial growth rates matched values reported in the same study. Likewise, a propionic acid-degrading population of *Syntrophobacter fumaroxidans* was simulated using model *i*Sfu648, in which NGAM is fixed to 0.14 mmol ATP gDW^-1^ h^-1^ (Hamilton et al., 2015). For butyric acid degradation, since no genome-scale model of known butyric acid-oxidizing bacteria is available yet, we augmented model *i*Sfu648 by the reactions catalyzed by the butyryl-CoA synthetase and the acetyl-CoA acetyltransferase, added butyric acid uptake via a proton symport, and enforced the excretion of 97.9% of the carbon influx as acetic acid, using the same ratio that the model predicted for growth on propionic acid. Parameter values for uptake kinetics were taken from Lawrence and McCarty (1969), resulting for propionic acid degradation in *S_in_* = 2.445 g L^-1^, *v_max_* = 3.538 g gDW^-1^ d^-1^, and *K_S_* = 31.85 mg L^-1^, and for butyric acid degradation in *S_in_* = 9.251 g L^-1^, *v_max_* = 10.53 g gDW^-1^ d^-1^, and *K_S_* = 5.022 mg L^-1^. Total community biomass was computed by summing up the biomass concentrations of the individual populations.

## Results and Discussion

### Experimental CSTR Performance

**FIGURE 2:**
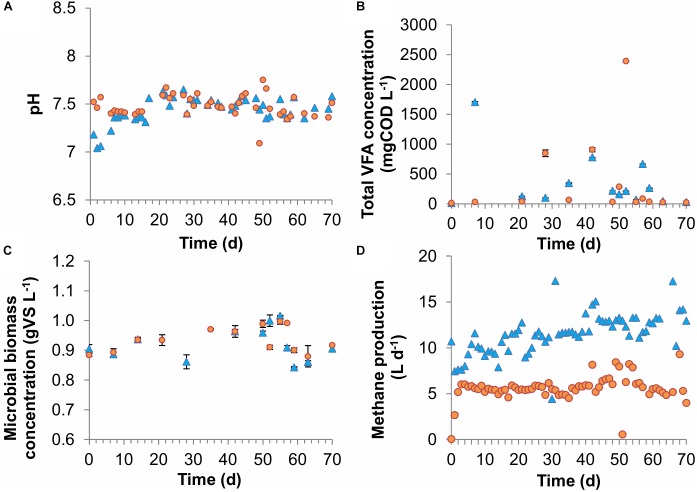
Performance of the lab-scale CSTRs. **(A)** pH, **(B)** VFA concentration, **(C)** microbial biomass concentration, and **(D)** methane production rate. The error bars represent 1 standard error of means.

In both reactors, the fed carbon sources acetic, propionic, and butyric acid were almost completely consumed throughout the experiment. Average VFA concentrations were low in both CSTRs with 337 ± 263 and 341 ± 383 mgCOD L^-1^ for the dilution rates of 0.18 days^-1^ and 0.07 days^-1^ [average ± standard error of mean (SEM)], respectively (Figure 2 and Supplementary Figures S1–S3), corresponding to substrate conversion efficiencies of >99% in both CSTRs. Low VFA concentrations even at high dilution rates have also been achieved before by Schmidt et al. (2014) who measured total VFA concentrations below 1000 mg L^-1^ in CSTRs fed with thin stillage with dilution rates as high as 0.33 days^-1^. Both CSTRs showed similar pH values with 7.44 ± 0.08 and 7.48 ± 0.07 for the dilution rates of 0.18 and 0.07 days^-1^, respectively (Figure 2). Methane production was close to its theoretical maximum of 6.2 L d^-1^, based on a complete conversion of COD in the influent to methane, for the CSTR with a dilution rate of 0.07 days^-1^, which corresponded to the high substrate conversion efficiencies (Figure 2). However, for the CSTR with a dilution rate of 0.18 days^-1^, the theoretical maximum of 14.2 L d^-1^ was only approached closely in the second half of the experiment, which can be explained by technical problems with the substrate feeding pump in the beginning of the experiment (Figure 2). However, this was not decisive for the overall experiment since the CSTR with a dilution rate of 0.18 days-^1^ ran stably for five HRTs in the second half and was therefore comparable to the CSTR with the dilution rate of 0.07 days^-1^, which also ran stably for five HRTs.

### Impact of Dilution Rate on Microbial Biomass Concentration

#### Strong Deviation of Experimental Results From All Models Using Default Parameters

Microbial biomass concentrations were almost equal for both CSTRs with average concentrations of 0.92 ± 0.03 and 0.94 ± 0.03 gVS L^-1^ for the dilution rates of 0.18 days^-1^ and 0.07 days^-1^, respectively (Figure 2). The average microbial biomass concentrations correspond to apparent microbial biomass yields for the total community of 24.5 and 25.0 mgVS gCOD_totalV FAs_^-1^ for the dilution rates of 0.18 and 0.07 days^-1^, respectively. Assuming an elemental biomass composition of CH_1.8_O_0.5_N_0.2_ (Kleerebezem and Van Loosdrecht, 2010), the apparent yields at both dilution rates were approximately 0.034 gCOD gCOD_totalV FAs_^-1^.

**FIGURE 3:**
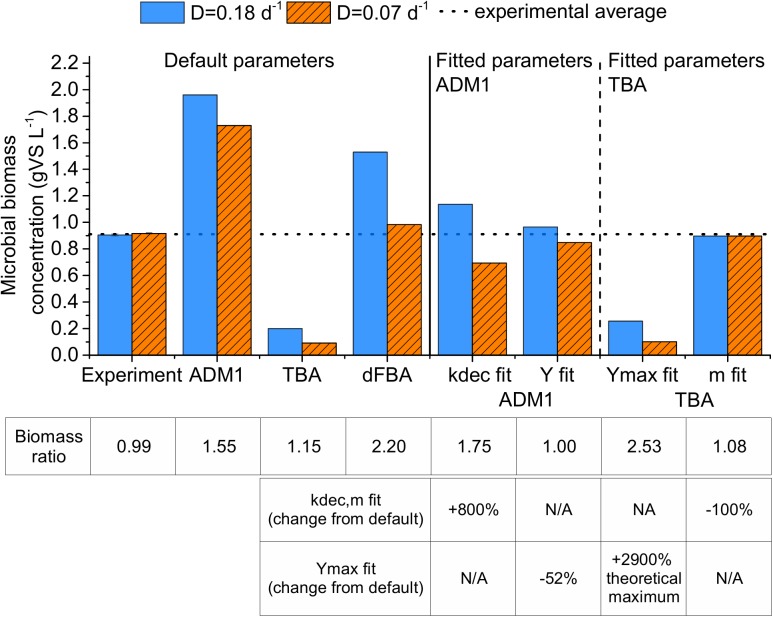
Experimentally observed microbial biomass concentrations and predictions by the three models: ADM1, Anaerobic Digestion Model 1; TBA, thermodynamic black box approach; and dFBA, dynamic Flux Balance Analysis; using default and fitted parameter values. Biomass ratio equals *X_D__=__*0.18*_/X_D__=__*0.07*_*. The error bars represent 1 standard error of means. N/A: not applicable.

These results were not predicted by the three models with their default parameter values (Figure 3). The predicted microbial biomass concentrations strongly exceeded the experimental results for the ADM1 and the dFBA, and strongly undercut the experimental results for the TBA. With an overestimation of total biomass concentration by 39.9% for the dilution rate of 0.18 days^-1^ and by 4.7% for the dilution rate of 0.07 days^-1^, the dFBA model provided the best match of all models using standard parameter values (see Supplementary Table S10).

All models predicted a higher biomass concentration at the dilution rate of 0.18 days^-1^, which was not observed in our experiment. This is supported by several other CSTR experiments that we conducted with the same substrate using dilution rates ranging between 0.07 and 0.18 days^-1^. Based on 139 samples in total from these experiments, we still did not find a significantly higher microbial biomass concentration at dilution rates of 0.18 days^-1^ compared to lower dilution rates (see Supplementary Figure S4, Supplementary Material A). Therefore, the difference between experimental and modeling results is likely caused by wrong model assumptions or an unsuitable parametrization.

#### Fitting the Models to the Experimental Results

We studied three ways of adjusting the TBA and ADM1 to fit the microbial biomass concentration: Either the parameter values for maintenance (*m, k_dec_*) or for biomass build-up (*Y_max_, Y*) were adjusted (see Figure 2). We refrained from fitting the dFBA model as here community biomass is computed as the sum resulting from four different models, hence providing many degrees of freedom for fitting.

In the ADM1 simulations, the default parameter values led to 91–123% higher microbial biomass concentrations than in the experiment (Figure 3, “ADM1”). The predicted microbial biomass concentration ratio (*X_D__=__*0.18*_*/*X_D__=__*0.07*_*) was 15% higher than in the experiment. Increasing *k_dec_* to fit the microbial biomass concentrations was therefore the wrong approach, because this further increased the microbial biomass concentration ratio error to 75% (Figure 3, “*k_dec_* fit”). Nevertheless, fitting ADM1 to experimental results by increasing *k_dec_* has been done several times. Weinrich (2017) reviewed ADM1 parameters from 30 publications. In this literature review, 20% of the studies used *k_dec_* values for acetogenesis and methanogenesis above 0.02 days^-1^ (see Supplementary Table S4, Supplementary Material A). Potentially, this is the result of overfitting in several studies because experiments comparing various dilution rates are scarce. Lawrence and McCarty (1969) operated several chemostats at dilution rates ranging between 0.083 and 0.667 days^-1^ at 35°C and they inferred an average decay rate of 0.016 ± 0.006 days^-1^. This supports our finding that *k_dec_* should not be increased above 0.02 days^-1^ to fit experimental results.

Reducing the default microbial biomass yields of ADM1 by 52% instead of increasing *k_dec_* led to good predictions of the microbial biomass concentration, which deviated by only 7–9% from the experimental values (see Figure 3, “Y fit”). These reduced microbial biomass yield parameter values also led to good predictions of a chemostat co-culture experiment with *Methanospirillum hungatei* and *Syntrophobacter fumaroxidans* fed with propionic acid at a dilution rate of 0.07 days^-1^ as described by Scholten and Conrad (2000). The apparent microbial biomass yield of this co-culture based on our fitted ADM1 microbial biomass yields is 0.025 gCOD_Xh2+Xpro_ gCOD_propionicacid_^-1^, which is close to their experimentally determined apparent yield of 0.021 gCOD_Xh2+Xpro_ gCOD_propionicacid_^-1^ (X_h2_ = *Methanospirillum hungatei*, X_pro_ = *Syntrophobacter fumaroxidans*, for details see Supplementary Material A, see section “ADM1 Simulation for Another Study on a Propionic Acid Fed Chemostat”). In comparison, the default ADM1 parameter values led to an overestimation of the apparent yield with a value of 0.051 gCOD_Xh2+Xpro_ gCOD_propionicacid_^-1^ for this co-culture experiment. This supports our finding that some of the default yield values in ADM1 might be in general too high. Nevertheless, all of the values assumed for the microbial biomass yields for acetogens and methanogens in the ADM1 studies reviewed by Weinrich (2017) were higher than the ones used to fit the experimental values in our study. In conclusion, for acetogenesis and methanogenesis in ADM1, values for *k_dec_* should be 0.02 days^-1^ or lower and values for microbial biomass yields should be about half of the default values by Rosen and Jeppsson (2006), if no experimental evidence for a direct determination of these parameter values is available.

Concerning the TBA, fitting only *Y_max_* was not sufficient to match the experimental results. A maximum microbial biomass concentration of 0.26 gVS L^-1^ could be reached this way, which was less than a third of the experimental microbial biomass concentration (Figure 3, “Y_max_ fit”). The reason was the high maintenance coefficient value. Setting the maintenance coefficient value to 0 kJ Cmol^-1^ h^-1^, i.e., no impact of dilution rate on apparent microbial biomass yield, the simulations deviated by less than 3% from the experimental microbial biomass concentrations (Figure 2, “mfit”). Scholten and Conrad (2000) reported similar findings with a methanogenic co-culture in a CSTR fed with propionic acid studying dilution rates in the range of 0.036–0.077 days^-1^ at 37°C. They inferred maintenance coefficients of 0.14–0.20 kJ Cmol^-1^ h^-1^ for the co-culture, almost two orders of magnitude below the maintenance coefficient of 9.8 kJ Cmol^-1^ h^-1^ suggested by Tijhuis et al. (1993). Similarly, Wandrey and Aivasidis (1983) determined a maintenance coefficient of 120 μmol_aceticacid_ g^-1^ h^-1^, corresponding to 0.09 kJ Cmol^-1^ h^-1^, for *Methanosarcina* in a CSTR fed with acetic acid using dilution rates in the range of 0.07–0.17 days^-1^ at 37°C. For our simulations, setting *m* to 0.20 instead of 0 kJ Cmol^-1^ h^-1^ and increasing *Y_max_* by 12% led the predicted microbial biomass concentrations to deviate by only 5% from the experimental values.

**FIGURE 4:**
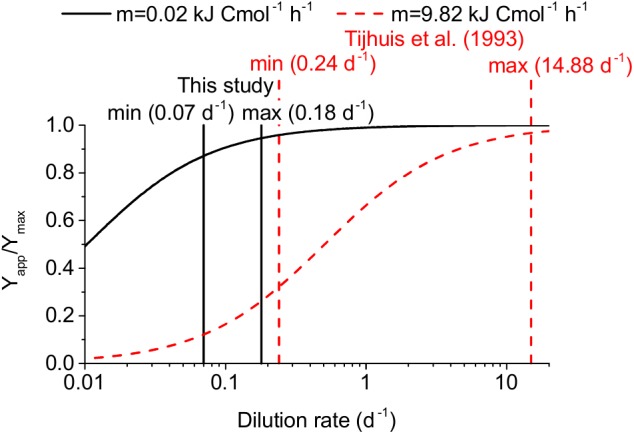
Impact of the dilution rate on the apparent yield for two maintenance coefficients: 0.02 kJ Cmol^-1^ h^-1^ recommended in our study for anaerobic digestion and 9.82 kJ Cmol^-1^ h^-1^ by Tijhuis et al. (1993). The minimum and maximum dilution rates used in the determination of these two values are indicated as vertical lines. The impact is expressed as apparent yield (Y_app_) divided by maximum yield (Y_max_).

Scholten and Conrad (2000) argued that the reason for such a much lower maintenance coefficient value might be that no syntrophs were included in the determination of the universal maintenance coefficient by Tijhuis et al. (1993). However, it was not clear why syntrophic VFA oxidizers appeared to be an exception to the otherwise universal maintenance coefficient. Therefore, we reanalyzed the studies that form the basis of this universal maintenance coefficient. We found that all of these studies applied higher dilution rates (0.24–14.88 days^-1^) compared to our study (0.07–0.18 days^-1^) and the other AD studies cited above (<0.17 days^-1^) (see Figure 4 and Supplementary Table S8). Therefore, the low maintenance coefficient we found might not only apply to acetogens and methanogens in anaerobic digestion but more generally to microorganisms growing at low dilution rates. This hypothesis is supported by Van Bodegom (2007) who found lower specific maintenance rates for microorganisms with lower maximum specific microbial growth rates based on a literature review.

A maintenance coefficient of 0.2 kJ Cmol h^-1^ corresponds to 0.116 mmol ATP gDW^-1^ h^-1^ assuming 70 kJ mol ATP^-1^ (Scholten and Conrad, 2000) and a microbial biomass composition of CH_1.8_O_0.5_N_0.2_ (Kleerebezem and Van Loosdrecht, 2010), which is lower than the default NGAM values in the employed FBA models ranging between 0.14 and 2 mmol ATP gDW^-1^ h^-1^. In conclusion, these literature values and our findings suggest that the maintenance coefficient value for acetogenesis and methanogenesis should be assumed to be smaller than 0.2 kJ Cmol^-1^ h^-1^ or 0.116 mmol ATP gDW^-1^ h^-1^ for dilution rates between 0.04 and 0.18 days^-1^ at 37°C, if no experimental evidence for a direct determination of this parameter value is available.

#### Transferability to Industrial Anaerobic Digesters

Determining the maintenance coefficient in industrial digesters is difficult. First, typical dilution rates can be as low as 0.01 days^-1^ (HRT > 100 days). Given that experiments should be run for at least three HRTs, an experiment would take at least 300 days, during which the substrate should remain the same. Second, it is almost impossible to separate microbial biomass from particulate matter being part of the substrate for an accurate quantification. Replacing the microbial biomass determination by molecular biological methods such as qPCR of 16S rRNA genes or microscopic methods would require a conversion of gene copy numbers or cell numbers to biomass. Such conversion factors would need to consider the number of 16S rRNA genes per genome, the number of genome copies per cell and the biomass per cell (Bonk et al., 2018). These information are not yet available for all species relevant for AD. Moreover, these parameters can vary not only between species but even within species (Soppa, 2014; Bonk et al., 2018).

Despite these uncertainties in using cell numbers instead of microbial biomasses, a maintenance coefficient of 9.8 kJ Cmol h^-1^ is still likely to result in a significant difference in cell numbers for greatly varying dilution rates. However, this did not apply to industrial anaerobic digesters studied by Krakat et al. (2010) and Nettmann et al. (2010). In both studies, the digester with the higher dilution rate should have resulted in 2.0–3.8 times higher microbial biomass yields as predicted by the TBA model with a maintenance coefficient of 9.8 kJ Cmol h^-1^ (see Table 1 and Supplementary Table S9. However, the higher dilution rates resulted in slightly lower cell yields (cell number per gCOD converted). As stated above, the use of cell numbers instead of microbial biomasses is problematic. Furthermore, the compared digesters did not run in parallel with identical substrate. Still, it seems likely that as in our experiments, the effect of dilution rate on microbial biomass is also lower in industrial digesters than expected by a high maintenance coefficient of 9.7 kJ Cmol h^-1^.

**Table 1:** Measured archaeal cell numbers vs. predicted biomass yields in digesters fermenting industrially relevant substrates at different dilution rates.

Experimental data (literature)				Prediction (this study)
	
Digester ID	Dilution rate (d^-1^)	Number of archaeal cells per gCOD VFA converted (cells gCODVFA-1)		Y_app_ (gCOD_X_ gCOD_V FA_^-1^) predicted for maintenance of 9.8 kJ Cmol^-1^ h^-1^
Nettmann et al., 2010				This study
R3	0.021	1.7⋅10^8^		1.2–3.0⋅10^-3^
R4	0.009	1.8⋅10^8^		3.3–8.5⋅10^-3^
**Ratio R3/R4**		**0.9**	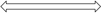	**3.6–3.8**
Krakat et al., 2010				This study
1600d	0.127	1.7⋅10^10^		6.9–13.4⋅10^-3^
650d	0.039	2.0⋅10^10^		2.5–5.7⋅10^-3^
**Ratio 1600d/650d**		**0.8**	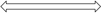	**2.4–2.8**


### Impact of Dilution Rate on Microbial Community Composition

**FIGURE 5:**
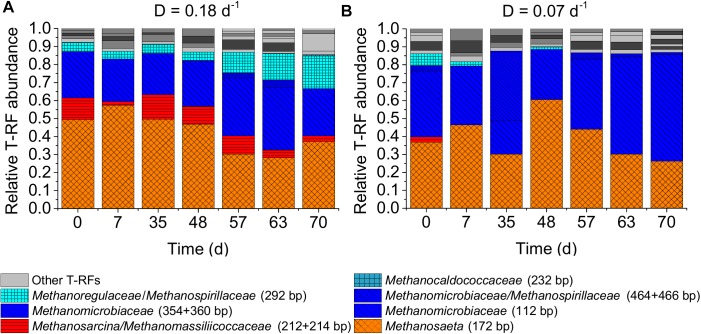
Methanogenic archaeal community dynamics in the two lab-scale CSTRs. T-RFLP profiles of *mcrA* amplicons digested with *Mwo*I for two reactors with dilution rates (D) of 0.18 days^-1^ **(A)** and 0.07 days^-1^ **(B)**. Only the assigned T-RFs are shown with T-RF length in bp in parentheses. Unassigned T-RFs are marked as gray solid bars.

Two factors could have contributed to the observed deviation of maintenance coefficient values reported by Tijhuis et al. (1993) and inferred in our study. First, Tijhuis et al. (1993) studied higher dilution rates and second, they used pure cultures. In microbial communities as used in our study, a decrease in dilution rate might lead to the enrichment of taxa evolutionary optimized for low microbial maintenance expenditure. However, the microbial communities of both CSTRs were in general quite similar concerning the dominant taxa at the end of the experiment. The methanogenic communities of both CSTRs were dominated by the hydrogenotrophic family *Methanomicrobiaceae* and the acetoclastic genus *Methanosaeta* during the whole experiment (Figure 5). This was confirmed in a T-RFLP analysis using the restriction enzyme *Bst*NI (Supplementary Figure S5, Supplementary Material A).

**FIGURE 6:**
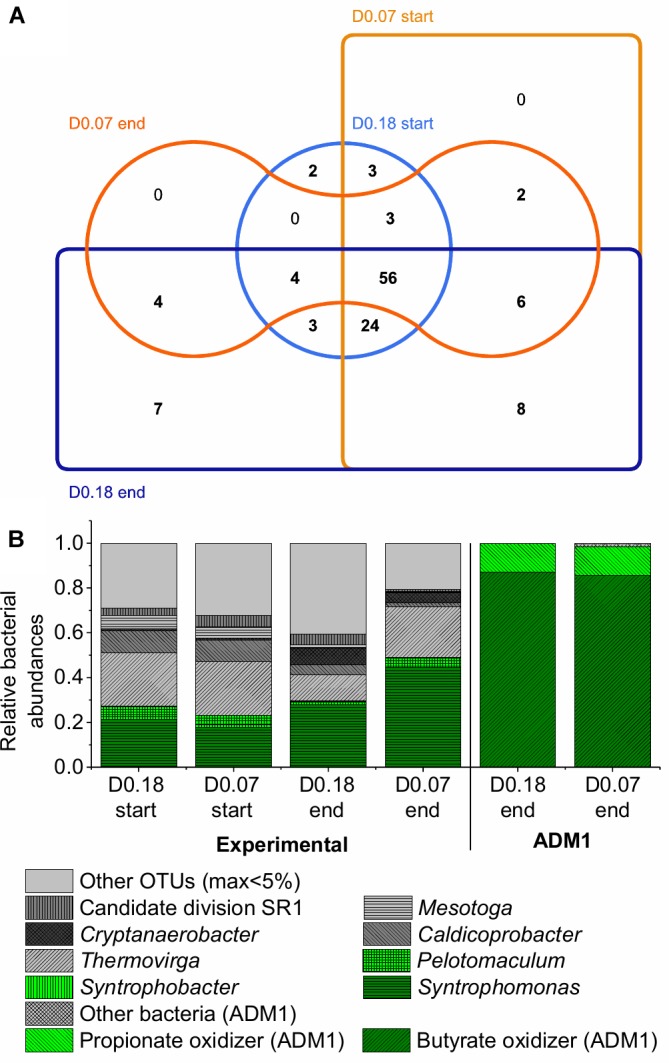
Bacterial community composition based on 16S rRNA amplicon sequencing at the start and end of the experiment for the two reactors with dilution rates of 0.18 days^-1^ (D0.18) and 0.07 days^-1^ (D0.07). Composition corrected for 16S rRNA gene copy number per genome. **(A)** Edwards–Venn diagram showing unique and shared OTUs between the samples visualized with jvenn (Bardou et al., 2014). **(B)** Relative OTU abundances compared to predictions by ADM1.

The bacterial communities also showed high similarities. At the end of the experiment, the two CSTRs shared 70 out of 112 OTUs for the dilution rate of 0.18 days^-1^ and out of 75 OTUs for the dilution rate 0.07 days^-1^ (Figure 6A and Supplementary Material C). Firmicutes were the most abundant bacterial reads on phylum level in both CSTRs with 53% and 62% of all bacterial reads for the dilution rates of 0.18 and 0.07 days^-1^, respectively. This phylum was dominated by the class Clostridia in both CSTRs (Supplementary Material C). The microbial community analysis showed two surprising results: the presence of *Methanosaeta* at high dilution rates as well as the presence of many bacterial OTUs not known to be involved in syntrophic VFA oxidation.

#### Unexpected Presence and Abundance of *Methanosaeta*

It is quite unexpected to find *Methanosaeta* dominating over *Methanosarcina* at a dilution rate as high as 0.18 days^-1^. Based on ADM1 simulations using experimentally derived kinetic parameters for *Methanosaeta* and *Methanosarcina* by Straub et al. (2006), *Methanosaeta* cannot sustain dilution rates higher than 0.11 days^-1^ and will be outcompeted at dilution rates higher than 0.07 days^-1^ under continuous feeding. Based on average kinetic parameters reported in the literature (Conklin et al., 2006), *Methanosaeta* is able to grow at a dilution rate of 0.18 days^-1^, but only at acetic acid concentrations above 2.4 g L^-1^ (see Supplementary Tables S5, S6). However, such high acetic acid concentrations were never measured in our experiment (see Supplementary Figure S1, Supplementary Material A). Furthermore, even if the acetic acid concentrations were higher than 2.4 g L^-1^, *Methanosarcina* would be predicted to dominate over *Methanosaeta* in a CSTR with a dilution rate of 0.18 days^-1^ (see Supplementary Material A, see section “Maximum Dilution Rate and Acetic Acid Concentration to Sustain Growth of Methanosaeta and Methanosarcina Based on Literature Kinetic Parameters”). However, at the end of our experiment, *Methanosaeta* reached relative abundances of 37% and 26% of total methanogens for the dilution rates of 0.18 and 0.07 days^-1^, respectively, while the abundance of *Methanosarcina* reached only 3% for the dilution rate of 0.18 days^-1^ and was not detectable for the dilution rate of 0.07 days^-1^.

Results contradicting reported kinetic growth parameters of *Methanosaeta* have been reported before. Ziganshin et al. (2016) found an increasing relative abundance of *Methanosaeta* when increasing the dilution rate of a CSTR from 0.33 to 0.67 days^-1^, which also should not be possible based on the reported parameters cited above.

The experimentally observed dominance of *Methanosaeta* over *Methanosarcina* is not reflected in the dFBA model, which only considers *Methanosarcina* as no FBA model for *Methanosaeta* is available yet. Between both these acetoclastic methanogens, the acetate activation mechanisms differs regarding the energy expenditure with *Methanosaeta* using ATP → AMP + PP_i_ and *Methanosarcina* using ATP → ADP + P_i_ (Welte and Deppenmeier, 2014). However, the energy conservation mechanisms of *Methanosaeta* are not fully understood yet (Berger et al., 2012). Nevertheless, the higher ATP demand for acetate activation of *Methanosaeta* will most likely lead to a lower maximum yield and thereby lower absolute biomass concentrations for *Methanosaeta* as observed previously (Conklin et al., 2006). Hence, our dFBA predictions for biomass define an upper boundary for the *Methanosarcina* dominated experimental community, and predicted differences in microbial biomass concentrations for different dilution rates will most likely remain unchanged.

#### Bacterial Community Composition

Although only acetic, propionic, and butyric acid were fed, less than half of the bacterial reads belonged to taxa known for the syntrophic oxidation of one of these acids (Figure 6B). *Syntrophobacter* and *Pelotomaculum* were found as the only known propionic acid-oxidizing genera. *Syntrophomonas* was found as the only known butyric acid-oxidizing genus. The higher abundance of butyric- than propionic acid-oxidizing bacteria can be attributed to the 4.5 times higher amount of butyric acid fed to the CSTRs (on COD basis).

The high abundance of bacteria not known to oxidize fatty acids could be explained by several reasons. First, there might be bacterial species present that are capable of, but not yet known for, syntrophic VFA oxidation. Secondly, bacteria could feed on decaying microbial biomass. However, based on ADM1 simulations using default parameters by Rosen and Jeppsson (2006), bacteria feeding on sugars, amino acids and fatty acids from decayed microbial biomass should make up at most 2% of total bacteria in our CSTRs, compared to an observed proportion of 51–77% of bacterial reads not known for VFA oxidation in the amplicon sequencing analysis (Figure 6B). Consumption of decaying biomass might thus be only a minor reason for the presence of bacteria other than the known VFA consumers.

Therefore, our microbial communities likely contain yet unknown VFA-oxidizing bacteria. This is further supported by the clearly lower abundance of *Syntrophomonas* at the dilution rate of 0.18 days^-1^ compared to the dilution rate of 0.07 days^-1^ (Figure 6B) although the same amount of butyric acid was consumed in both CSTRs. Therefore, it seems worthwhile to analyze the community by meta-omics and cultivation techniques for novel syntrophic VFA degraders. A literature survey (Supplementary Table S2, Supplementary Material A) provides first hints on which of the taxa might be responsible for VFA oxidation and which ones might be responsible for degradation of dead microbial biomass. Cloacimonetes (Pelletier et al., 2008; Juste-Poinapen et al., 2015; Ahlert et al., 2016), *Cryptanaerobacter* (de Bok et al., 2005), and *Desulfovibrio* (Suzuki et al., 2010) have been connected with propionic or butyric acid oxidation but no co-culture of one of their species with a hydrogenotrophic methanogen has been tested yet. *Candidatus* Cloacamonas acidaminovorans, for example, contains all genes for propionic acid oxidation via methylmalonyl-CoA but has not been isolated yet (Pelletier et al., 2008).

## Conclusion

The impact of dilution rate, and thereby specific microbial growth rate, on the apparent microbial biomass yield has been introduced as a maintenance coefficient or decay rate in various models with severe consequences for predicted microbial biomass concentrations.

The default parameter values of maintenance for the three studied models ADM1, TBA, and dFBA led to overestimated differences in predicted microbial biomass concentrations between the two dilution rates studied in our experiments. A low impact of specific microbial growth rate on apparent yield could also be found in several chemostat studies using VFAs as sole carbon source. Therefore, we concluded that the maintenance coefficient in the TBA should be chosen to be below 0.2 kJ Cmol h^-1^, corresponding to 0.116 mmol ATP gDW^-1^ h^-1^, and the decay rate in ADM1 should be chosen to be below 0.016 days^-1^ for acetogenesis and methanogenesis as the default at 37°C, if no experimental evidence for a direct determination of this parameter value is available. For the maintenance coefficient, this is almost two orders of magnitude below the default value suggested for the TBA in the literature.

The reason for this strong difference might lie in the low dilution rates applied in our study and AD studies in general, compared to the high dilution rates used to derive the universal maintenance coefficient. Potentially, slow growing microorganisms might have in general lower maintenance coefficients. If so, our findings for AD might similarly apply to other engineered and natural microbial processes running at low dilution rates. Such systems include for example the production of VFAs from primary sludge from wastewater treatment plants [D∼0.25 days^-1^ (Pittmann and Steinmetz, 2013)], the cow rumen [D∼0.25 days^-1^ (Abe and Kumeno, 1973)], medium chain fatty acid production from organic wastes [D∼0.11 days^-1^ (Kucek et al., 2016)] or the deep-sea bed [growth rates smaller 0.004 days^-1^ (Jørgensen and Boetius, 2007)].

## Data Availability Statement

The datasets generated for this study can be found in the Supplementary Material B and the EMBL European Nucleotide Archive (ENA) under accession number PRJEB22603 (see text http://www.ebi.ac.uk/ena/data/view/PRJEB22603).

## Author Contributions

FB, HS, SK, and FC designed the reactor experiments. SW implemented the ADM1 model and contributed to the discussion of the simulation results. FB performed the reactor experiments, T-RFLP data analysis, and simulations for the TBA and ADM1. DP and DB performed the 16S rRNA gene sequencing data analysis. FC performed the dFBA simulations. HH contributed to the discussion of the results. All authors prepared, read, and approved the manuscript.

## Conflict of Interest Statement

The authors declare that the research was conducted in the absence of any commercial or financial relationships that could be construed as a potential conflict of interest.

## Footnotes

1 http://bbmap.sourceforge.net/

2 http://www.ebi.ac.uk/ena/data/view/PRJEB22603

3 https://rrndb.umms.med.umich.edu/
