# Supplementary material for: Determination of Microbial Maintenance in Acetogenesis and Methanogenesis by Experimental and Modeling Techniques
Source: Front Microbiol. 2019 Feb 8;10:166. doi: 10.3389/fmicb.2019.00166 (PMC6375858; doi:10.3389/fmicb.2019.00166)
Supplement: Supplementary file 1 [file Data_Sheet_1.zip › Supplementary Materials/Supplementary Material A.pdf]

Supplementary Material A

Determination of microbial maintenance in acetogenesis and methanogenesis by experimental and modelling techniques

Fabian Bonk*, Denny Popp, Sören Weinrich, Heike Sträuber, Daniela Becker, Sabine Kleinsteuber, Hauke Harms, Florian Centler

*** Correspondence:**
Corresponding Author
fabian.bonk@ufz.de

1 Composition of the mineral medium

The synthetic medium consisted of two components (component A and component B, equal volume) which were kept separated until feeding to avoid precipitation.

Table S1. Composition of the mineral medium

Compound	Concentration in partitioned media (mg L^{-1})	Final concentration in CSTR influent (mg L^{-1})
	Component A	
Acetic acid	31,420	15,710
Propionic acid	4,920	2,460
Butyric acid	18,440	9,220
KCl	600	300
$MgCl_2 \times 6H_2O$	600	300
$CaCl_2 \times 2H_2O$	200	100
$Na_2S \times 9H_2O$	500	250
$FeCl_2 \times 4H_2O$	42.36	21.18
$CuCl_2 \times 2H_2O$	0.86	0.43
$CoCl_2 \times 6H_2O$	1.94	0.97

**Compound**	**Concentration in partitioned media (mg L^{-1})**	**Final concentration in CSTR influent (mg L^{-1})**
$MnCl_2 \times 4H_2O$	1.64	0.82
$Na_2MoO_4 \times 2H_2O$	0.86	0.43
$NiCl_2 \times 6H_2O$	3.28	1.64
$Na_2WO_4 \times 2H_2O$	0.36	0.18
$Na_2SeO_3 \times 5H_2O$	0.8	0.40
$ZnCl_2$	4.72	2.36
H_3BO_3	4.96	2.48
Biotin	0.04	0.02
Folic acid	0.04	0.02
Pyridoxine	0.2	0.1
Thiamine	0.1	0.05
Riboflavin	0.1	0.05
Nicotinic acid	0.1	0.05
Ca-Pantothenate	0.1	0.05
Vitamin B12	0.1	0.05
p-Aminobenzoate	0.1	0.05
Lipoic acid	0.1	0.05
	Component B	
KH_2PO_4	1000	500
NH_4HCO_3	8190	4095
NaOH	5600	2800

2 CSTR performance

2.1 Acetic, propionic, and butyric acid concentrations in the CSTRs

Acetic, propionic, and butyric acid concentrations for both CSTRs are shown in Figure S1, S2, and S3, respectively.

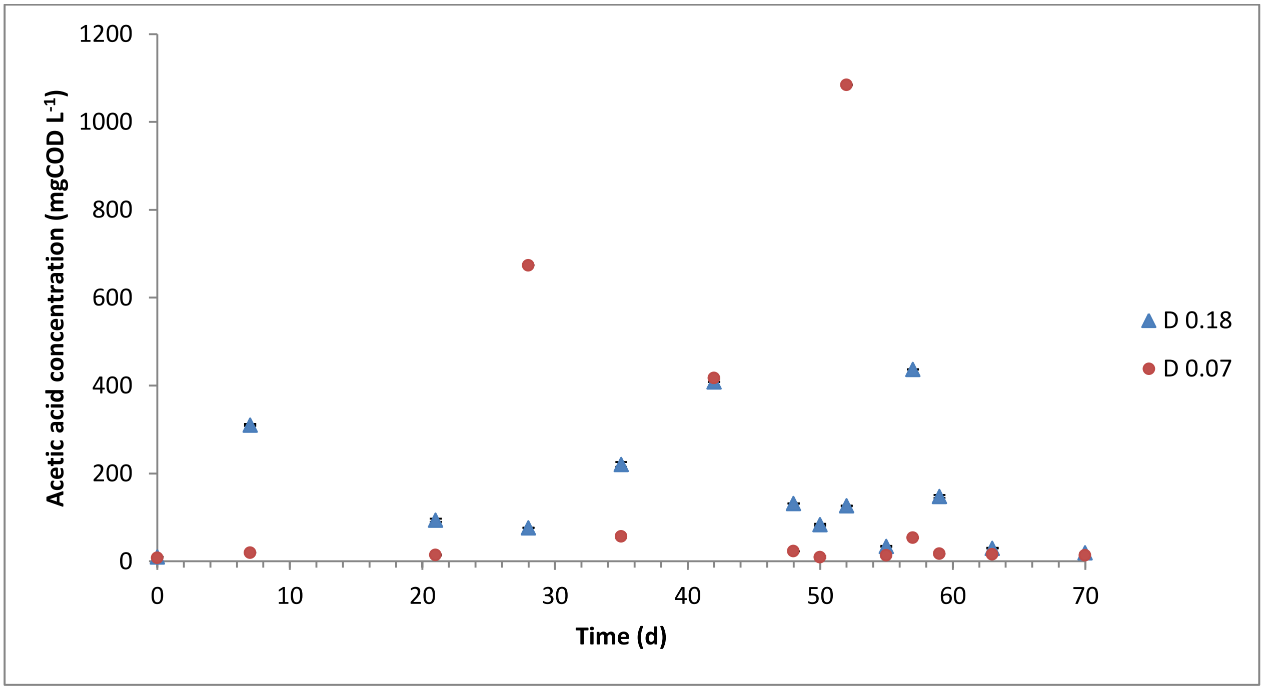

Figure S1. Acetic acid concentrations in the two CSTRs with the dilution rates of 0.18 d^{-1} and 0.07 d^{-1}. Error bars indicate 1 standard error of means (SEM).

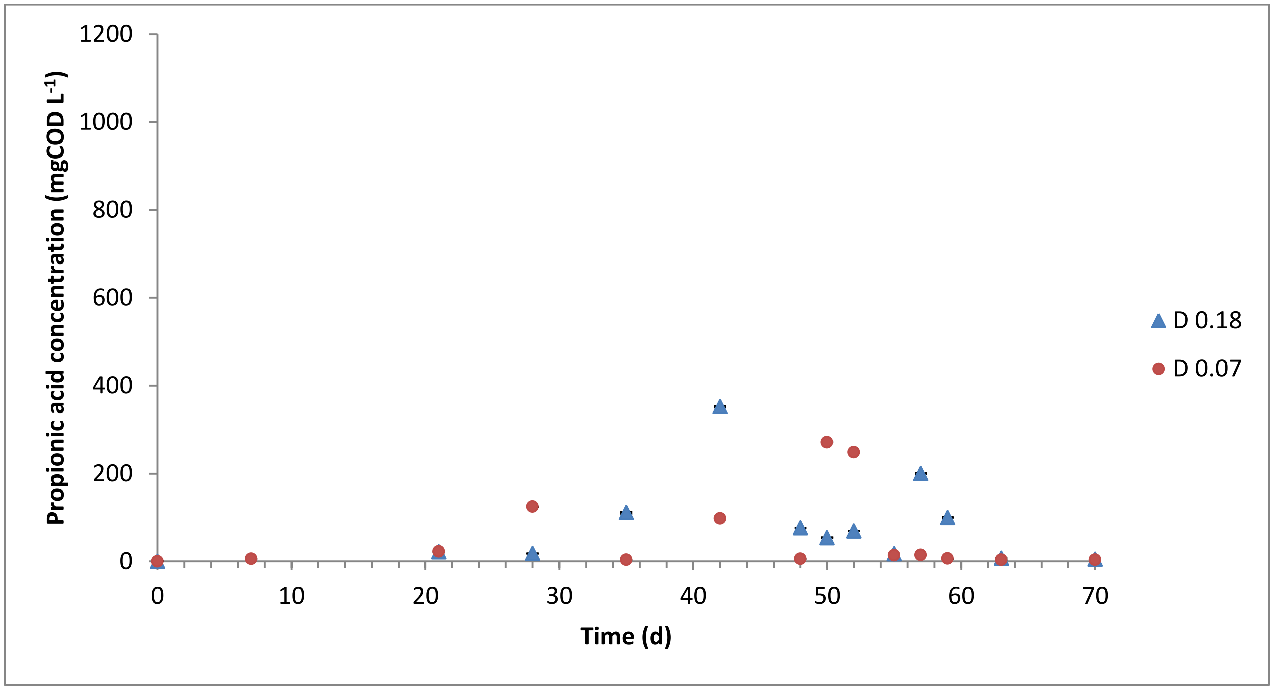

Figure S2. Propionic acid concentrations in the two CSTRs with the dilution rates of 0.18 d^{-1} and 0.07 d^{-1}. Error bars indicate 1 standard error of means (SEM).

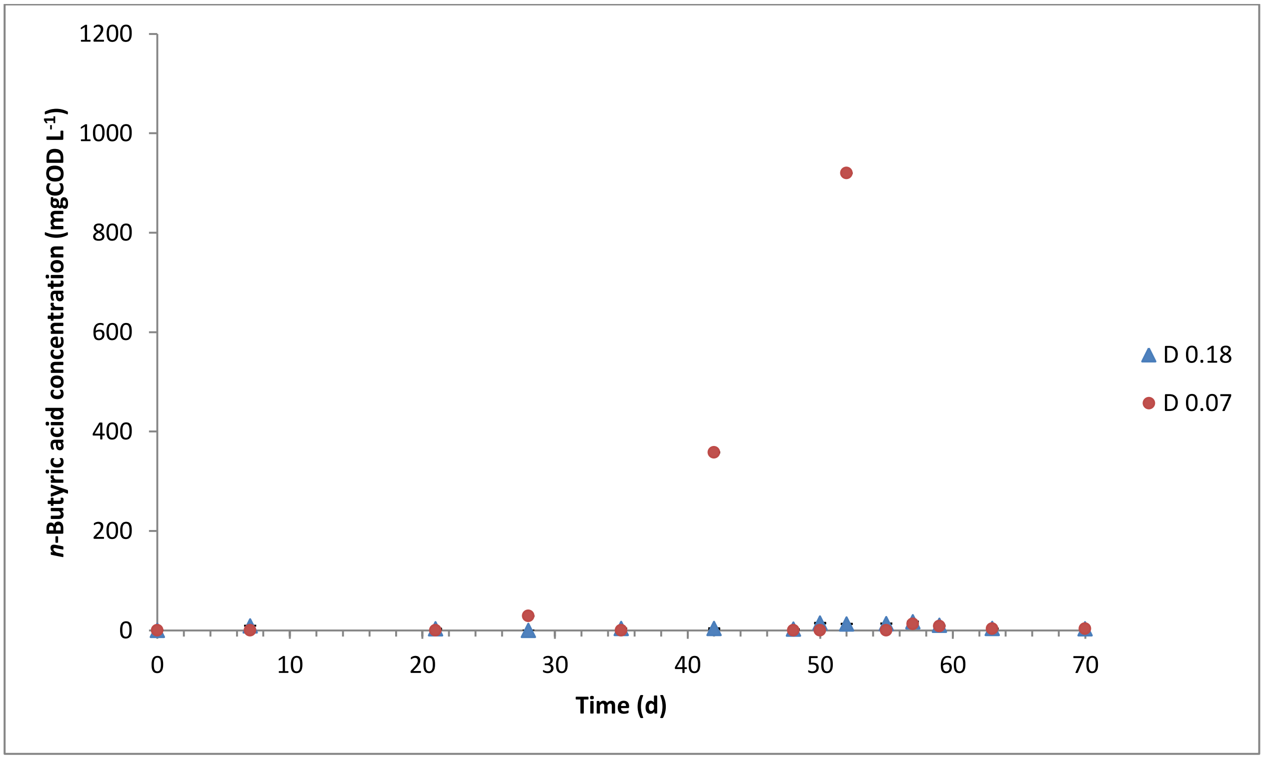

Figure S3. *n*-Butyric acid concentrations in the two CSTRs with the dilution rates of 0.18 d^{-1} and 0.07 d^{-1}. Error bars indicate 1 standard error of means (SEM).

2.2 Microbial biomass concentrations from several other of our experiments

Biomass concentrations from several CSTR experiments which we conducted under the same conditions as described in the manuscript but not with an identical but similar inoculum are shown in Figure S4. All samples were taken after at least 3 HRTs of continuous operation.

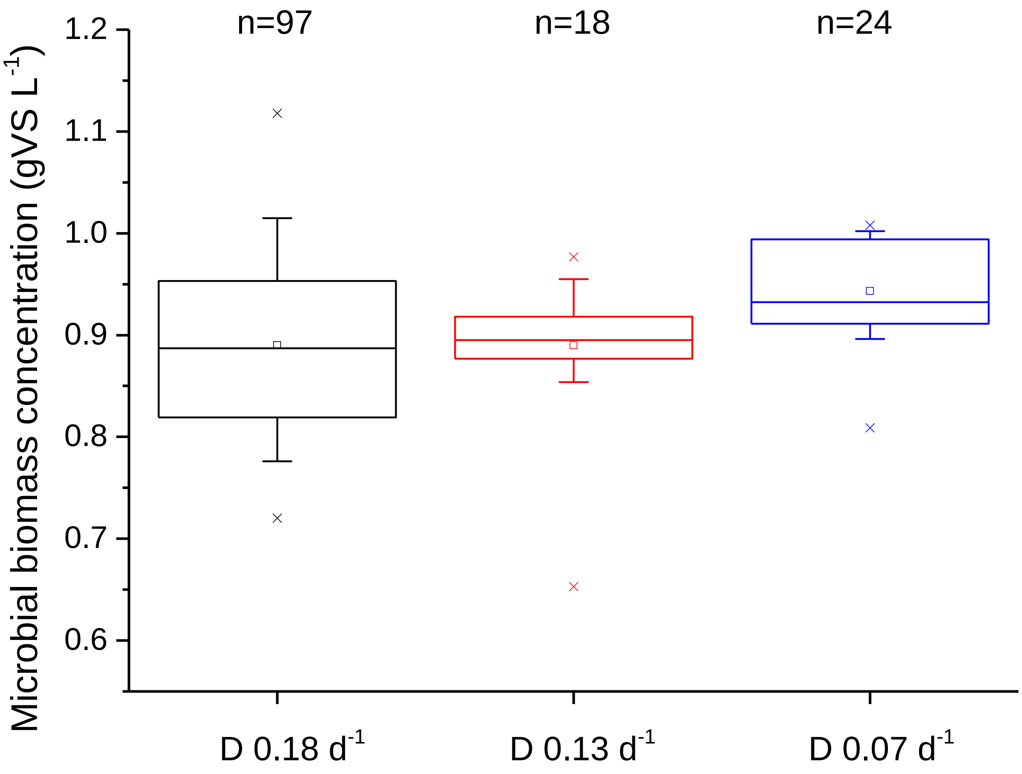

Figure S4. Microbial biomass concentration measurements after at least 3 HRTs of continuous operation from various experiments we conducted using three different dilution rates (0.18, 0.13, and 0.07 d^{-1}). Whiskers represent the 2nd and 3rd quartile, the band in the middle the median, the square in the middle the average, and the × minimum and maximum values.

3 Microbial community structure

3.1 T-RFLP analysis of *mcrA* genes with restriction enzyme *Bst*NI

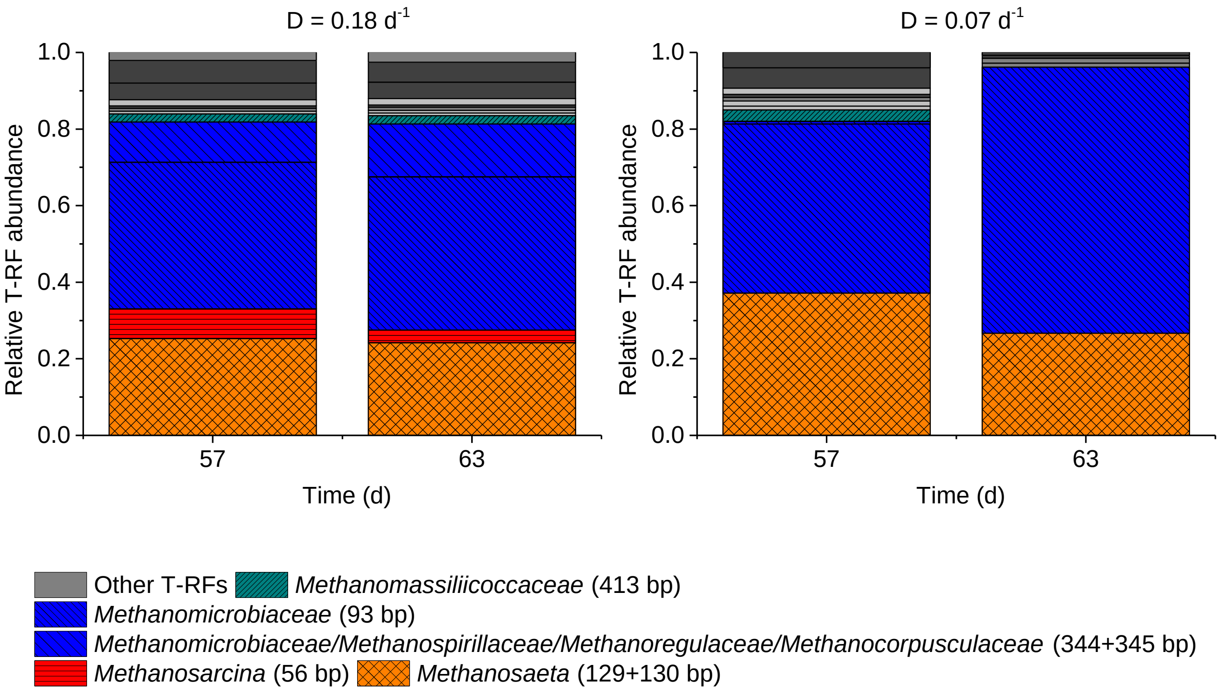

Figure S5. Community dynamics of methanogenic archaea in the two lab scale CSTRs operated at dilution rates of 0.18 d^{-1} and 0.07 d^{-1}. T-RFLP profiles of *mcrA* amplicons digested with *Bst*NI. Only the assigned T-RFs are presented with T-RF lengths in parentheses. Unassigned T-RFs are marked as grey solid bars.

3.2 Bacterial community composition

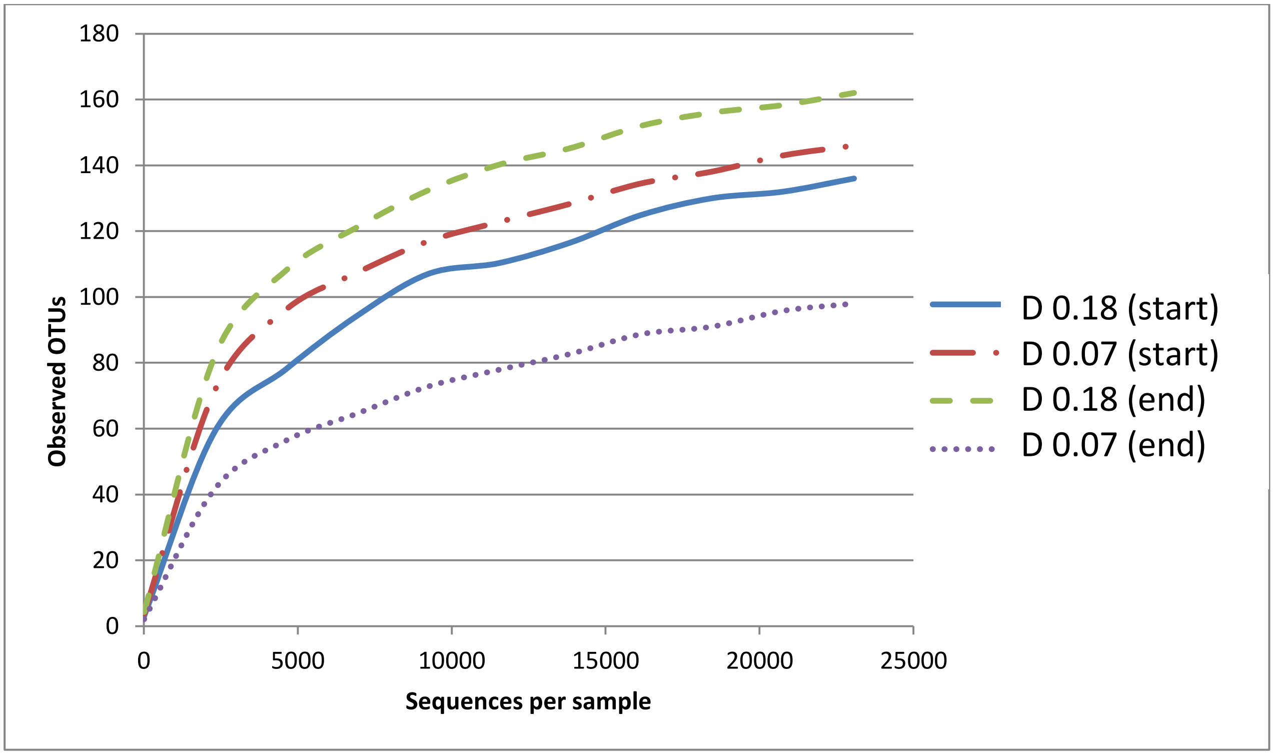

Figure S6. Rarefaction curves of the samples from both CSTRs for the dilution rates 0.18 d^{-1} (D 0.18) and 0.07 d^{-1} (D 0.07) at the beginning and end of the experiment.

3.3 Physiological information on the most bacterial taxa

Table S2. Physiological information on the most abundant bacterial taxa with a focus on syntrophic propionic acid and butyric acid oxidation

Genus or higher level if not available	Physiological characterization and noteworthy occurrence in anaerobic digesters (high abundance, correlation with process parameters) of the closest relative
Thermovirga	*Thermovirga lienii*: amino acids fermenter under thermophilic conditions (Dahle and Birkeland, 2006)
Syntrophomonas	Known for syntrophic butyric acid oxidation (Sousa et al., 2007)
Caldicoprobacter	*Caldicoprobacter oshimai*: thermophilic, xylanolytic, sugar fermenting (Yokohama et al., 2010). *Caldicoprobacter* spp. dominated a mesophilic anaerobic digester with total ammonia nitrogen conc. of 25 g/L (Poirier et al., 2016).
Mesotoga	*Mesotoga prima*: growth on sugars and amino acids (Nesbø et al., 2012)
Syntrophobacter	Known for syntrophic propionic acid oxidation (Boone and Bryant, 1980)
Pelotomaculum	Known for syntrophic propionic acid oxidation (Imachi et al., 2002; de Bok et al., 2005)
Sphingobacteriales	*Sphingobacteriales* spp. dominant in high solid AD of rape straw (Tian et al., 2017)
Spirochaetaceae	*Spirochaetaceae* were suggested to be involved in methanogenic hexadecane degradation as inferred by DNA-SIP (Cheng et al., 2013)
Gelria	*Gelria glutamica*: thermophilic, obligate syntrophic glutamate degrader. Growth on carbohydrates. Growth on amino acids in co-culture with *Methanobacterium thermautotrophicum*, but not on acetic or propionic acid. Butyric acid not tested (Balk et al., 2002). *Gelria* spp. were suggested to be capable of acetic acid degradation as inferred by protein-SIP assay (Mosbaek et al., 2016).
Candidate division SR1	Detected in anaerobic digester sludge but not dominant (Hao et al., 2016)
Cloacimonetes	*Candidatus* Cloacamonas acidaminovorans contains all genes for propionic acid oxidation via methylmalonyl-CoA (Pelletier et al., 2008). *Cloacamonas* spp. not tested in co-culture but connected to propionic acid oxidation in other studies (Juste-Poinapen et al., 2015), (Ahlert et al., 2016)
Peptococcaceae	*Pelotomaculum* belonging to this family is known for syntrophic oxidation of propionic acid (de Bok et al., 2005; Imachi et al., 2002)
Aminivibrio	*Aminivibrio pyruvatiphilus*: growth on amino acids and organic acids, but not on propionic or butyric acid in co-culture (Honda et al., 2013).
Sedimentibacter	*Sedimentibacter* spp. were involved in the degradation of accumulated VFAs in an anaerobic digester fed with pig manure, fish waste and molasses residues (Regueiro et al., 2014).
Cryptanaerobacter	*Cryptanaerobacter phenolicus* is the closest relative found to the obligate syntrophic propionic acid oxidizer *Pelotomaculum schinkii* but has not been tested yet for propionic acid or butyric acid oxidation (de Bok et al., 2005).
Desulfovibrio	*Desulfovibrio butyratiphilus*: isolated from an anaerobic municipal sewage sludge digester. Butyric acid oxidizing and sulfate reducing bacterium (Suzuki et al., 2010). *Desulfovibrio vulgaris* capable of syntrophic lactate degradation in co-culture with *Methanosarcina* (Plugge et al., 2011).
Ruminococcaceae	*Ruminococcaceae* spp. abundant in an agitated anaerobic digester with accumulated propionic acid (Tian et al., 2014)
Blvii28 wastewater-sludge group	*Acetobacteroides hydrogenigenes*: growth on carbohydrates, but not on propionic acid or butyric acid in co-culture with *Methanospirillum hungatei* (McIlroy et al., 2015)

4 Theoretical considerations

4.1 ADM1

4.1.1 Impact of biomass recycling

Table S3. Effect of microbial biomass recycling on total microbial biomass X_{total} in ADM1in steady state

	Dilution rate	
	**0.18 d^{-1}**	**0.07 d^{-1}**
X_{total} (gCOD L^{-1})	2.683	2.368
X_{total} without microbial biomass recycling X_{total} (gCOD L^{-1})	2.678	2.340
Increase in X_{total} by microbial biomass recycling	0.18%	1.21%
$\Sigma(X_{ac}, X_{pro}, X_{C4}, X_{h2})$ (gCOD L^{-1})	2.680	2.352
$\Sigma(X_{ac}, X_{pro}, X_{C4}, X_{h2})$ without microbial biomass recycling X_{total} (gCOD L^{-1})	2.678	2.340
Increase in $\Sigma(X_{ac}, X_{pro}, X_{C4}, X_{h2})$ by microbial biomass recycling	0.08%	0.54%

Based on simulation results in steady state conditions for $f_{ch,xc} = 0.2$, $f_{pr,xc} = 0.2$, $f_{li,xc} = 0.3$, $f_{sI,xc} = 0.1$, $f_{xI,xc} = 0.2$, $k_{dec} = 0.02$ d^{-1} and $k_{dis} = 0.5$ d^{-1} (Rosen and Jeppsson, 2006).

4.1.2 Derivation of Equation 2(apparent yield Y_{app} for ADM1)

The ordinary differential equations for biomass built up in ADM1 follow Equation S1.

$$\frac{dX}{dt} = Y \cdot k_m \cdot \frac{S}{S+K_S} \cdot I \cdot X - k_{dec} \cdot X - X \cdot D + X_{in} \cdot D \text{ (Equation S1)}$$

with X being the microbial biomass in the reactor, X_{in} the microbial biomass in the influent, D the dilution rate, Y the maximum microbial biomass yield, k_m the specific maximum specific substrate uptake rate, S the substrate, I various inhibition terms, K_S the half-saturation constant and k_{dec} the microbial decay rate.

The ordinary differential equations for substrate consumption in ADM1 follow Equation S2.

$$\frac{dS}{dt} = -X \cdot k_m \cdot \frac{S}{S+K_S} \cdot I - S \cdot D + S_{in} \cdot D + \dot{S}_i \text{ (Equation S2)}$$

with X being the microbial biomass in the reactor, D the dilution rate, S_{in} the substrate concentration in the influent, $\dot{S}_i$ the substrate production rate from other reactions (e.g. acetic acid production in butyric acid oxidation), I various inhibition terms, k_m the specific maximum specific substrate uptake rate, S the substrate concentration in the reactor, and K_S the half-saturation constant.

Assuming steady state and by replacing X in Equation S2 with $Y_{app} \cdot \left(S_{in} + \frac{\dot{S}_i}{D} - S\right)$, Equation S3 can be derived:

$$Y_{app} = \frac{(S+K_S) \cdot D}{S \cdot k_m \cdot I} \text{ (Equation S3)}$$

Assuming steady state for Equation S1 and X_{in}=0, Equation S4 can be derived:

$$\frac{S+K_S}{I \cdot S \cdot k_m} = \frac{Y}{k_{dec}+D} \text{ (Equation S4)}$$

By combining Equation S3 and S4, an expression for Y_{app} can be derived:

$$Y_{app} = \frac{Y}{\frac{k_{dec}}{D}+1} \text{ (Equation 2)}$$

4.1.3 Summary of studies with high k_{dec} values

Table S4. Summary of studies with values for k_{dec} for acetic acid, propionic acid, butyric acid and hydrogen degraders in ADM1 greater than 0.02 d^{-1} based on an analysis by Weinrich (2017)

Population	k_{dec}	Reference
X_{pro}	0.05	(Dereli et al., 2010)
X_{pro}	0.04	(Girault et al., 2011)
X_{pro}	0.10	(Lübken, 2009)
X_{pro}	0.04	(Page et al., 2008)
X_{pro}	0.06	(Zhou et al., 2011)
X_{c4}	0.05	(Dereli et al., 2010)
X_{c4}	0.04	(Girault et al., 2011)
X_{c4}	0.10	(Lübken, 2009)
X_{c4}	0.04	(Page et al., 2008)
X_{c4}	0.06	(Schoen et al., 2009)
X_{c4}	0.06	(Zhou et al., 2011)
X_{ac}	0.05	(Dereli et al., 2010)
X_{ac}	0.04	(Girault et al., 2011)
X_{ac}	0.10	(Lübken, 2009)
X_{ac}	0.13	(Schoen et al., 2009)
X_{ac}	0.06	(Zhou et al., 2011)
X_{h2}	0.05	(Dereli et al., 2010)
X_{h2}	0.04	(Girault et al., 2011)
X_{h2}	0.10	(Lübken, 2009)
X_{h2}	0.30	(Schoen et al., 2009)
X_{h2}	0.06	(Zhou et al., 2011)

4.1.4 Maximum dilution rate and acetic acid concentration to sustain growth of *Methanosaeta* and *Methanosarcina* based on literature kinetic parameters

Based on the microbial growth equation of the original ADM1 (Batstone et al., 2002) but without microorganisms in the influent and no inhibition, the concentration change of *Methanosaeta* or *Methanosarcina* in a CSTR follows:

$$\frac{dX}{dt} = Y \cdot k_m \cdot \frac{S}{S+K_S} \cdot X - X \cdot (k_{dec} + D) \quad \text{(Equation S5)}$$

with endogenous decay rate k_{dec}, half-saturation constant K_S, microbial biomass concentration X, dilution rate D, yield Y, acetic acid concentration S and the maximum specific acetic acid uptake rate k_m.

For $S >> K_S$ and no other microorganisms present that compete for acetic acid, the maximum dilution rate (D_{max}) that sustains *Methanosaeta* or *Methanosarcina* in a CSTR in steady state can be calculated by rearranging Equation S5:

$$D_{max} = (Y \cdot k_m - k_{dec}) \quad \text{(Equation S6)}$$

Likewise, the minimum acetic acid concentration (S_{min}) that sustains *Methanosaeta* or *Methanosarcina* can be calculated as:

$$S_{min} = K_S \cdot (k_{dec} + D) / \big(Y \cdot k_m - (k_{dec} + D)\big) \quad \text{(Equation S7)}$$

Conklin et al. (2006) calculated the average of kinetic growth parameters for *Methanosaeta* from five scientific papers. Straub et al. (2006) also published kinetic growth parameters for *Methanosaeta* based on experimental data. These parameters and the resulting S_{min} and D_{max} values are presented in Table S5. Note that the average literature values calculated by Conklin et al. (2006) did not contain the k_{dec} value. Therefore, it was set to 0.0064 d^{-1} based on Straub et al. (2006).

Conklin et al. (2006) calculated the average of kinetic growth parameters for *Methanosarcina* based on eleven scientific papers. Straub et al. (2006) also published kinetic growth parameters for *Methanosarcina* based on experimental data. These parameters and the resulting S_{min} and D_{max} are presented in Table S6. Note that the average literature values calculated by Conklin et al. (2006) did not contain the k_{dec} value. Therefore, it was set to 0.1 d^{-1} based on Straub et al. (2006).

Table S5. Growth kinetic parameters for *Methanosaeta* and maximum dilution rates and substrate concentrations to sustain *Methanosaeta* in a CSTR

**Average literature values calculated by Conklin et al. (2006)**		**Based on experimental data calculated by Straub et al. (2006)**	
Y (mgCOD mgCOD^{-1})*	0.026	Y (mgCOD mgCOD^{-1})	0.042
K_S (mgCOD L^{-1})	49	K_S (mgCOD L^{-1})	90
k_m (mgCOD mgCOD^{-1} d^{-1})*	7.39	k_m (gCOD gCOD^{-1} d^{-1})	2.77
		k_{dec} (d^{-1})	0.0064
Calculated based on literature values		Calculated based on literature values	
D_{max} (d^{-1})	0.19	D_{max} (d^{-1})	0.11
S_{min} for D=0.18 d^{-1} (mgCOD L^{-1})	2505		No growth possible at D=0.18 d^{-1}
S_{min} for D=0.18 d^{-1} (mg L^{-1})	2351		

* converted from mass to COD base assuming 1.366 gCOD gVSS^{-1} (Kleerebezem and Van Loosdrecht, 2010)

Table S6. Growth kinetic parameters for *Methanosarcina* and resulting maximum dilution rates and substrate concentrations to sustain *Methanosarcina* in a CSTR

**Average literature value calculated by Conklin et al. (2006)**		**Straub et al. (2006) based on experimental data**	
Y (mgCOD mgCOD^{-1})*	0.066	Y (mgCOD mgCOD^{-1})	0.06
K_S (mgCOD L^{-1})	280	K_S (mgCOD L^{-1})	320
k_m (mgCOD mgCOD^{-1} d^{-1})*	8.93	k_m (gCOD gCOD^{-1} d^{-1})	8.95
		k_{dec} (1/d)	0.1
Calculated based on literature values		Calculated based on literature values	
D_{max} (d^{-1})	0.48	D_{max} (d^{-1})	0.44
S_{min} for D=0.18 d^{-1} (mgCOD L^{-1})	260	S_{min} for D=0.18 d^{-1} (mgCOD L^{-1})	353
S_{min} for D=0.18 d^{-1} (mg L^{-1})	244		

* converted from mass to COD base assuming 1.366 gCOD gVSS^{-1} (Kleerebezem and Van Loosdrecht, 2010)

4.1.5 ADM1 simulation for another study on a propionic acid fed chemostat

Scholten and Conrad (2000) measured an average apparent yield of 15 mgVS $gCOD^{-1}$ (no flushing of the headspace with N_2/CO_2, biomass dry weight calculated from measured protein concentration) in a CSTR containing a co-culture of *Methanospirillum hungatei* and *Syntrophobacter fumaroxidans* fed with propionic acid at a concentration of 4.48 gCOD L^{-1} and a dilution rate of 0.07 d^{-1}. This apparent yield corresponds to 21 mgCOD $gCOD_{propionic\ acid}^{-1}$ assuming an elemental biomass composition of $CH_{1.8}O_{0.5}N_{0.2}$ (Kleerebezem and Van Loosdrecht, 2010). Note that the co-culture did not contain an acetoclastic methanogen or syntrophic acetic acid oxidizing bacteria. Using the parameter values of the ADM1 "Y fit" scenario (Figure 3), the simulation of the co-culture described above in ADM1 results in an apparent yield of 25 mgCOD $gCOD_{propionic\ acid}^{-1}$, which is much closer to the experimental results of Scholten and Conrad (2000) than the default ADM1 parameter values, which result in an apparent yield of 0.051 mgCOD $gCOD_{propionic\ acid}^{-1}$.

4.2 Thermodynamic black box approach (TBA)

4.2.1 Details on the approach used in this study

Heijnen (1999) reported an Arrhenius type of equation for maintenance energy demand m_G [$\frac{kJ}{Cmol_X \cdot h}$] that is only dependent on temperature T and independent of carbon or nitrogen source as well as electron donor or acceptor (Equation S8a) with X being the microbial biomass. This correlation was found to be accurate within a range of ± 40% over a wide range of different microorganisms, temperatures ranging between 5-75°C (Heijnen, 2002). Equation S5a is the average of a correlation for aerobic (Equation S8b) and anaerobic (Equation S8c) microorganisms by Tijhuis et al. (1993). For our study, we used Equation S8c to calculate the maintenance energy of 9.82 kJ Cmol h^{-1} for 37°C.

$$m_G = 4.5 \cdot \exp\left(-\frac{69000}{R} \cdot \left(\frac{1}{T} - \frac{1}{298}\right)\right) \text{ (Equation S8a)}$$

$$m_G = 5.7 \cdot \exp\left(-\frac{69000}{R} \cdot \left(\frac{1}{T} - \frac{1}{298}\right)\right) \text{ (Equation S8b)}$$

$$m_G = 3.3 \cdot \exp\left(-\frac{69000}{R} \cdot \left(\frac{1}{T} - \frac{1}{298}\right)\right) \text{ (Equation S8c)}$$

Here, R is the ideal gas constant and T [K] the temperature of the CSTR.

By dividing the maintenance energy rate m_G by the catabolic energy ΔG^{01} (Table S7), the maintenance related substrate consumption rate m [$\frac{mol_S}{Cmol_X \cdot h}$] can be obtained:

$$m = \frac{m_G}{\Delta G01} \text{ (Equation S9)}$$

Here, the standard Gibbs energy corrected for a pH of 7 for the conversion of acetic acid, propionic acid, and butyric acid to methane was used to estimate the impact of maintenance energy. To get more accurate results, the Gibbs energy should be corrected for the prevailing environmental conditions in each time step in a dynamic model. This was omitted to avoid further complexity. The difference between standard Gibbs energy at pH 7 and corrected Gibbs energy was calculated to be less than 5% given the environmental conditions at the end of the experiment. Therefore the chosen approach should give a reasonable estimate. Note that propionic acid and butyric acid are first oxidized to acetic acid, H_2 and CO_2, and in a second step, H_2 and CO_2 are converted to methane. A hypothetical direct conversion to CH_4 was used because it avoids the inaccuracies and complexity of correcting for hydrogen partial pressure, which has a crucial impact on the catabolic Gibbs energy.

Y_{max} values based on the Gibbs energy dissipation method and Gibbs energy changes of catabolism were taken from Table A5 presented by Kleerebezem and Van Loosdrecht (2010), see Table S7.

Table S7. Maximum yields and Gibbs energy change of catabolism used for the TBA with CO_2 as electron acceptor

**Electron donor**	**Y_{max} ($Cmol_X$ mol_S^{-1})**	**Standard Gibbs energy change of catabolism at pH 7 ΔG^{01} (kJ mol^{-1})**
Acetic acid	0.06	-31.1
Propionic acid	0.12	-56.2
Butyric acid	0.16	-81.6

4.2.2 Experimental basis for the universal maintenance coefficient

Equation S8b was derived by Tijhuis et al. (1993) from the chemostat studies summarized in Table S8. The dilution rates used are much higher than those used in our study and higher than the maximum dilution rate found for methane fermentation of VFAs of 0.37 d^{-1} (Lawrence and McCarty, 1969). Interestingly, there is also one batch study with recycling of biomass in the analysis of Tijhuis et al. (1993). This study of Arbige and Chesbro (1982) about the growth of *Bacillus polymyxa*, a maintenance coefficient of 0 was found for specific growth rates between 0.005 and 0.05 h^{-1}, which correspond to the specific growth rates at steady state for mixed culture chemostat anaerobic digestion for HRTs of 0.8-8.3 d. However, Tijhuis et al. (1993) did not use this value of 0 in their analysis but a value of 5.8 kJ Cmol h^{-1}, which was indirectly derived from diphenylamine reactive, orcinol positive, and Lowry positive compounds by Arbige and Chesbro (1982).

Table S8. Dilution rates of the chemostat studies that are the basis for the universal maintenance coefficient compared to dilution rates used in anaerobic digestion

**Microorganism**	**Dilution rate (h^{-1})**	**References**
**Tijhuis et al. (1993)**		
Acetogenium kivui	0.04-0.45	(von Eysmondt et al., 1990)
Aerobacter aerogenes	0.07-0.6	(Stouthamer and Bettenhaussen, 1975)
Bacteroides amylophilus	0.03-0.4	(Jenkinson and Woodbine, 1979)
Campylobacter sp.	0.02-0.2	(Laanbroek and Veldkamp, 1979)
Clostridium butyricum	0.07-0.4	(Crabbendam et al., 1985)
Desulfovibrio vulgaris	0.05-0.23	(Nethe-Jaenchen and Thauer, 1984)
Klebsiella aerogenes	0.1-0.62	(Streekstra et al., 1987)
Methanobacterium formicium	0.03-0.08	(Chua and Robinson, 1981)
Methanobacterium thermoautotrophicum	0.08-0.2	(Fardeau et al., 1987)
Methanococcus thermolithotrophicus	0.07-0.13	(Fardeau et al., 1987)
Microbacterium thermosphactum	0.02-0.1	(Rogers et al., 1980)
Saccharomyces cerevisiae	0.03-0.19	(Rogers and Stewart, 1974)
Zymomonas	0.01-0.14	(Fieschko and Humphrey, 1996)
**Mixed culture anaerobic digestion**		
Max. dilution rate	0.015	(Lawrence and McCarty, 1969)
This study	0.003-0.008	This study

4.2.3 Details on measured archaeal cell numbers versus predicted biomass yields in digesters fermenting industrially relevant substrates at different dilution rates

Since microbial biomass concentrations are difficult to determine in digesters fed with particulate substrate, literature information on archaeal cell numbers determined by fluorescent in-situ hybridization (FISH) was used instead. Such a comparison can only give a first impression and no accurate relationship for several reasons. First, average microbial biomass for the archaeal cells might vary between the digesters. Secondly, the contribution of the individual VFAs, hydrogen and other intermediates to the methane production rate is difficult to determine. Here, we calculated scenarios for methane production from either acetic, propionic or butyric acid.

Table S9 Details on measured archaeal cell numbers versus predicted biomass yields in digesters fermenting industrially relevant substrates at different dilution rates

**Experimental data from the literature**							**Prediction (This study)**		
Digester ID	Substrate	Dilution rate (d^{-1})	Methane production rate ($m^3\ m^{-3}\ d^{-1}$)	Number of archaeal cells (cells L^{-1})	Estimated VFA conversion based on methane production rate (gCOD L^{-1})[a]	Number of archaeal cells per gCOD VFA converted (cells $gCOD_{VFA}^{-1}$)	Y_{app} ($gCOD_X\ gCOD_{VFA}^{-1}$) predicted for maintenance of 9.8 kJ $Cmol^{-1}\ h^{-1}$<br>Acetic acid	Propionic acid	Butyric acid
			**Nettmann et al. (2010)**				**This study**		
R3	Pig liquid manure (50%), maize silage (40%)	0.021	1.3	$3.0 \cdot 10^{10}$	176	$1.7 \cdot 10^{8}$	$1.2 \cdot 10^{-3}$	$2.2 \cdot 10^{-3}$	$3.0 \cdot 10^{-3}$
R4	Maize silage (82%), barley grain (12%)	0.009	1.4	$7.9 \cdot 10^{10}$	432	$1.8 \cdot 10^{8}$	$3.3 \cdot 10^{-4}$	$5.9 \cdot 10^{-4}$	$8.5 \cdot 10^{-4}$
					**Ratio R3/R4**	**0.9** ⇔	**3.8**	**3.7**	**3.6**
			**Krakat et al. (2010)**				**This study**		
1600d	Fodder beet silage	0.127	5.3	$2.0 \cdot 10^{12}$	119	$1.7 \cdot 10^{10}$	$6.9 \cdot 10^{-3}$	$1.1 \cdot 10^{-2}$	$1.3 \cdot 10^{-2}$
650d	Fodder beet silage	0.039	1.9	$2.9 \cdot 10^{12}$	144	$2.0 \cdot 10^{10}$	$2.5 \cdot 10^{-3}$	$4.2 \cdot 10^{-3}$	$5.7 \cdot 10^{-3}$
					**Ratio 1600d/650d**	**0.8** ⇔	**2.8**	**2.6**	**2.4**

[a] Assuming methane is solely produced from VFAs, 0.35 L methane $gCOD_{VFA}$, and neglecting microbial biomass build-up.

4.3 Detailed simulation results for the dFBA models

The biomass and substrate concentration in steady state for the individual dFBA simulations are shown in Table S10.

Table S10. Steady state biomass and substrate concentrations of the dFBA simulations

	**Biomass (gDW L^{-1})**		**Substrate effluent conc. (mg L^{-1})**	
	D 0.18 d^{-1}	D 0.07 d^{-1}	D 0.18 d^{-1}	D 0.07 d^{-1}
Acetoclastic methanogenesis	0.89	0.45	461.693	266.270
Hydrogenotrophic methanogenesis	0.14	0.09	0.011	0.007
Butyric acid oxidation	0.32	0.32	5.110	1.234
Propionic acid oxidation	0.18	0.12	64.449	20.742

References

Ahlert, S., Zimmermann, R., Ebling, J., and König, H. (2016). Analysis of propionate-degrading consortia from agricultural biogas plants. *Microbiologyopen* 5, 1027–1037. doi:10.1002/mbo3.386.

Arbige, M., and Chesbro, W. R. (1982). Very slow growth of *Bacillus polymyxa*: Stringent response and maintenance energy. *Arch. Microbiol.* 132, 338–344. doi:10.1007/BF00413386.

Balk, M., Zoetendal, E. G., Plugge, C. M., and Stams, A. J. M. (2002). *Gelria glutamica* gen. nov., sp. nov., a thermophilic, obligately syntrophic, glutamate-degrading anaerobe. *Int. J. Syst. Evol. Microbiol.* 52, 401–407. doi:10.1099/00207713-52-2-401.

Batstone, D. J., Keller, J., Angelidaki, I., Kalyuzhnyi, S. V., Pavlostathis, S. G., Rozzi, A., et al. (2002). *Anaerobic Digestion Model No.1. (ADM1)*. London: IWA Publishing.

Boone, D. R., and Bryant, M. P. (1980). Propionate-Degrading Bacterium , *Syntrophobacter wolinii* sp . nov . gen . nov ., from Methanogenic Ecosystems. *Appl. Environ. Microbiol.* 40, 626–632.

Cheng, L., Ding, C., Li, Q., He, Q., Dai, L. R., and Zhang, H. (2013). DNA-SIP Reveals That *Syntrophaceae* Play an Important Role in Methanogenic Hexadecane Degradation. PLoS One 8, 1–11. doi:10.1371/journal.pone.0066784.

Chua, H. B., and Robinson, J. P. (1981). Formate-limited growth of *Methanobacterium formicium* in steady-state cultures. *Enzyme* 6, 216– 220. doi:10.3747/pdi.2011.00058.

Conklin, A., Stensel, H. D., and Ferguson, J. (2006). Growth kinetics and competition between *Methanosarcina* and *Methanosaeta* in mesophilic anaerobic digestion. *Water Environ. Res.* 78, 486–496. doi:10.2175/106143006X95393.

Crabbendam, P. M., Neijssel, O. M., Tempest, D. W., and Van Amsterdam, U. (1985). Metabolic and energetic aspects of the growth of *Clostridium butyricum* on glucose in chemostat culture. *Arch. Microbiol.* 142, 375–382.

Dahle, H., and Birkeland, N. K. (2006). *Thermovirga lienii* gen. nov., sp. nov., a novel moderately thermophilic, anaerobic, amino-acid-degrading bacterium isolated from a North Sea oil well. *Int. J. Syst. Evol. Microbiol.* 56, 1539–1545. doi:10.1099/ijs.0.63894-0.

De Bok, F. A. M., Harmsen, H. J. M., Plugge, C. M., de Vries, M. C., Akkermans, A. D. L., de Vos, W. M., et al. (2005). The first true obligately syntrophic propionate-oxidizing bacterium, *Pelotomaculum schinkii* sp. nov., co-cultured with Methanospirillum hungatei, and emended description of the genus Pelotomaculum. *Int. J. Syst. Evol. Microbiol.* 55, 1697–1703. doi:10.1099/ijs.0.02880-0.

Dereli, R. K., Ersahin, M. E., Ozgun, H., Ozturk, I., and Aydin, A. F. (2010). Applicability of Anaerobic Digestion Model No. 1 (ADM1) for a specific industrial wastewater: Opium alkaloid effluents. *Chem. Eng. J.* 165, 89–94. doi:10.1016/j.cej.2010.08.069.

Fardeau, M. L., Peillex, J. P., and Belaïch, J. P. (1987). Energetics of the growth of *Methanobacterium thermoautotrophicum* and *Methanococcus thermolithotrophicus* on ammonium chloride and dinitrogen. *Arch. Microbiol.* 148, 128–131. doi:10.1007/BF00425360.

Fieschko, J., and Humphrey, A. E. (1996). Effects of temperature and ethanol concentration on the maintenance and yield coefficient of zymomonas mobilis. *Biotechnol. Bioeng.* 25, 1655–1660.

Girault, R., Rousseau, P., Steyer, J. P., Bernet, N., and Béline, F. (2011). Combination of batch experiments with continuous reactor data for ADM1 calibration: Application to anaerobic digestion of pig slurry. *Water Sci. Technol.* 63, 2575–2582. doi:10.2166/wst.2011.594.

Hao, L., Bize, A., Conteau, D., Chapleur, O., Courtois, S., Kroff, P., et al. (2016). New insights into the key microbial phylotypes of anaerobic sludge digesters under different operational conditions. *Water Res.* 102, 158–169. doi:10.1016/j.watres.2016.06.014.

Heijnen, J. J. (2002). Bioenergetics of microbial growth. In: Encyclopedia of Bioprocess Technology, eds. M. C. Flickinger and S. W. Drew (Hoboken, NJ, USA: John Wiley & Sons, Inc.), 267–291. doi:10.1002/0471250589.ebt026.

Honda, T., Fujita, T., and Tonouchi, A. (2013). *Aminivibrio pyruvatiphilus* gen. nov., sp. nov., an anaerobic, amino-acid-degrading bacterium from soil of a Japanese rice field. *Int. J. Syst. Evol. Microbiol.* 63, 3679–3686. doi:10.1099/ijs.0.052225-0.

Imachi, H., Sekiguchi, Y., Kamagata, Y., Hanada, S., Ohashi, A., and Harada, H. (2002). *Pelotomaculum thermopropionicum* gen. nov., sp. nov., an anaerobic, thermophilic, syntrophic propionate-oxidizing bacterium. *Int. J. Syst. Evol. Microbiol.* 52, 1729–1735. doi:10.1099/ijs.0.02212-0.

Jenkinson, H. F., and Woodbine, M. (1979). Growth and energy production in *Bacteroides amylophilus*. *Arch. Microbiol.* 120, 275–281.

Juste-Poinapen, N. M. S., Turner, M. S., Rabaey, K., Virdis, B., and Batstone, D. J. (2015). Evaluating the potential impact of proton carriers on syntrophic propionate oxidation. *Sci. Rep.* 5, 18364. doi:10.1038/srep18364.

Kleerebezem, R., and Van Loosdrecht, M. C. M. (2010). A generalized method for thermodynamic state analysis of environmental systems. *Crit. Rev. Environ. Sci. Technol.* 40, 1–54. doi:10.1080/10643380802000974.

Krakat, N., Schmidt, S., and Scherer, P. (2010). Mesophilic fermentation of renewable biomass: Does hydraulic retention time regulate methanogen diversity. *Appl. Environ. Microbiol.* 76, 6322–6326. doi:10.1128/AEM.00927-10.

Laanbroek, H. J., and Veldkamp, H. (1979). Growth yield and energy generation in anaerobically-grown *Campylobacter* spec. *Arch. Microbiol.* 120, 47–51. doi:10.1007/BF00413272.

Lawrence, A. W., and McCarty, P. L. (1969). Kinetics of methane fermentation in anaerobic treatment. *Water Pollut. Control Fed.* 41, 1–17. doi:10.2307/25036255.

Lübken, M. (2009). Mathematical modeling of anaerobic digestion processes. Dissertation. Technical University of Munich, Munich, Germany.

McIlroy, S. J., Saunders, A. M., Albertsen, M., Nierychlo, M., McIlroy, B., Hansen, A. A., et al. (2015). MiDAS: the field guide to the microbes of activated sludge. *Database* 2015, bav062. doi:10.1093/database/bav062.

Mosbæk, F., Kjeldal, H., Mulat, D. G., Albertsen, M., Ward, A. J., Feilberg, A., et al. (2016). Identification of syntrophic acetate-oxidizing bacteria in anaerobic digesters by combined protein-based stable isotope probing and metagenomics. *ISME J.* 10, 2405–2418.

Nesbø, C. L., Bradnan, D. M., Adebusuyi, A., Dlutek, M., Petrus, A. K., Foght, J., et al. (2012). *Mesotoga prima* gen. nov., sp. nov., the first described mesophilic species of the Thermotogales. *Extremophiles* 16, 387–393. doi:10.1007/s00792-012-0437-0.

Nethe-Jaenchen, R., and Thauer, R. K. (1984). Growth yields and saturation constant of *Desulfovibrio vulgaris* in chemostat culture. *Arch. Microbiol.* 137, 236–240.

Nettmann, E., Bergmann, I., Pramschufer, S., Mundt, K., Plogsties, V., Herrmann, C., et al. (2010). Polyphasic analyses of methanogenic archaeal communities in agricultural biogas plants. *Appl. Environ. Microbiol.* 76, 2540–2548. doi:10.1128/AEM.01423-09.

Page, D. I., Hickey, K. L., Narula, R., Main, A. L., and Grimberg, S. J. (2008). Modeling anaerobic digestion of dairy manure using the IWA Anaerobic Digestion Model no. 1 (ADM1). *Water Sci. Technol.* 58, 689. doi:10.2166/wst.2008.678.

Pelletier, E., Kreimeyer, A., Bocs, S., Rouy, Z., Gyapay, G., Chouari, R., et al. (2008). "*Candidatus* Cloacamonas Acidaminovorans": Genome Sequence Reconstruction Provides a First Glimpse of a New Bacterial Division. *J. Bacteriol.* 190, 2572–2579. doi:10.1128/JB.01248-07.

Poirier, S., Desmond-Le Quéméner, E., Madigou, C., Bouchez, T., and Chapleur, O. (2016). Anaerobic digestion of biowaste under extreme ammonia concentration: Identification of key microbial phylotypes. *Bioresour. Technol.* 207, 92–101. doi:10.1016/j.biortech.2016.01.124.

Plugge, C. M., Zhang, W., Scholten, J. C. M., and Stams, A. J. M. (2011). Metabolic flexibility of sulfate-reducing bacteria. *Front. Microbiol.* 2, 1–8. doi:10.3389/fmicb.2011.00081.

Regueiro, L., Carballa, M., and Lema, J. M. (2014). Outlining microbial community dynamics during temperature drop and subsequent recovery period in anaerobic co-digestion systems. *J. Biotechnol.* 192, 179–186. doi:10.1016/j.jbiotec.2014.10.007.

Rogers, P. J., and Stewart, P. R. (1974). Energetic efficiency and maintenance energy characteristics of *Saccharomyces cerevisiae* (wild type and petite) and *Candida parapsilosis* grown aerobically and micro-aerobically in continuous culture. *Arch. Microbiol.* 99, 25–46. doi:10.1007/BF00696220.

Rogers, P. J., Taylor, V. K., and Egan, A. F. (1980). Energetics of growth of *Microbacterium thermosphactum* at low temperatures. *Arch. Microbiol.* 128, 152–156. doi:10.1007/BF00406152.

Rosen C, Jeppsson U .(2006). Aspects on ADM1 implementation within the BSM2 Framework, Technical Report, Department of Industrial Electrical Engineering and Automation (IEA). Lund University, Lund, Sweden.

Schoen, M. A., Sperl, D., Gadermaier, M., Goberna, M., Franke-Whittle, I., Insam, H., et al. (2009). Population dynamics at digester overload conditions. *Bioresour. Technol.* 100, 5648–5655. doi:10.1016/j.biortech.2009.06.033.

Scholten, J. C. M., and Conrad, R. (2000). Energetics of syntrophic propionate oxidation in defined batch and chemostat cocultures. *Appl. Environ. Microbiol.* 66, 2934–2942. doi:10.1128/AEM.66.7.2934-2942.2000.

Sousa, D. Z., Smidt, H., Madalena Alves, M., and Stams, A. J. M. (2007). *Syntrophomonas zehnderi* sp. nov., an anaerobe that degrades long-chain fatty acids in co-culture with Methanobacterium formicicum. *Int. J. Syst. Evol. Microbiol.* 57, 609–615. doi:10.1099/ijs.0.64734-0.

Stouthamer, A. H., and Bettenhaussen, C. W. (1975). Determination of the efficiency of oxidative phosphorylation in continuous cultures of *Aerobacter aerogenes*. *Arch. Microbiol.* 102, 187–192. doi:10.1007/BF00428367.

Straub, A. J., Conklin, A. S. Q., Ferguson, J. F., and Stensel, H. D. (2006). Use of the ADM1 to investigate the effects of acetoclastic methanogen population dynamics on mesophilic digester stability. *Water Sci. Technol.* 54, 59–66. doi:10.2166/wst.2006.526.

Streekstra, H., Teixeira de Mattos, M. J., Neijssel, O. M., and Tempest, D. W. (1987). Overflow metabolism during anaerobic growth of *Klebsiella aerogenes* NCTC 418 on glycerol and dihydroxyacetone in chemostat culture. *Arch. Microbiol.* 147, 268–275. doi:10.1007/BF00463487.

Suzuki, D., Ueki, A., Shizuku, T., Ohtaki, Y., and Ueki, K. (2010). *Desulfovibrio butyratiphilus* sp. nov., a Gram-negative, butyrate-oxidizing, sulfate-reducing bacterium isolated from an anaerobic municipal sewage sludge digester. *Int. J. Syst. Evol. Microbiol.* 60, 595–602. doi:10.1099/ijs.0.013771-0.

Tian, Z., Cabrol, L., Ruiz-Filippi, G., and Pullammanappallil, P. (2014). Microbial Ecology in Anaerobic Digestion at Agitated and Non-Agitated Conditions. *PLoS One* 9, e109769. doi:10.1371/journal.pone.0109769.

Tian, J. H., Pourcher, A. M., Bureau, C., and Peu, P. (2017). Cellulose accessibility and microbial community in solid state anaerobic digestion of rape straw. *Bioresour. Technol.* 223, 192–201. doi:10.1016/j.biortech.2016.10.009.

Tijhuis, L., Van Loosdrecht, M. C. M., and Heijnen, J. J. (1993). A thermodynamically based correlation for maintenance Gibbs energy requirements in aerobic and anaerobic chemotrophic growth. *Biotechnol. Bioeng.* 42, 509–519. doi:10.1002/bit.260420415.

Von Eysmondt, J., Vasic-Racki, D., and Wandrey, C. (1990). Acetic acid production by *Acetogenium kivui* in continuous culture - kinetic studies and computer simulations. *Appl. Microbiol. Biotechnol.* 34, 344–349. doi:10.1007/BF00170056.

Weinrich, S. (2017). Praxisnahe Modellierung von Biogasanlagen: Systematische Vereinfachung des Anaerobic Digestion Model No. 1 (ADM1). Dissertation.University of Rostock, Rostock, Germany.

Yokohama, H., Wagner, I. D., and Wiegel, J. (2010). *Caldicoprobacter oshimai* gen. nov., sp. nov., an anaerobic, xylanolytic, extremely thermophilic bacterium isolated from sheep faeces, and proposal of *Caldicoprobacteraceae* fam. nov. *Int. J. Syst. Evol. Microbiol.* 60, 67–71. doi:10.1099/ijs.0.011379-0.

Zhou, H., Löffler, D., and Kranert, M. (2011). Model-based predictions of anaerobic digestion of agricultural substrates for biogas production. *Bioresour. Technol.* 102, 10819–10828. doi:10.1016/j.biortech.2011.09.014.
