# Supplementary material for: Determination of Microbial Maintenance in Acetogenesis and Methanogenesis by Experimental and Modeling Techniques
Source: Front Microbiol. 2019 Feb 8;10:166. doi: 10.3389/fmicb.2019.00166 (PMC6375858; doi:10.3389/fmicb.2019.00166)
Supplement: Supplementary file 1 [file Data_Sheet_1.zip › Supplementary Materials/Supplementary Materials C.html]

Javascript must be enabled to view this page.

magnitude
score


D 0.18 d start
D 0.07 d start
D 0.18 d end
D 0.07 d end

 1
 1
 1
 1

-.457221825409149
-.537482125493544
-.408725714134624
-2.23722937473523E-02

 1
 1
 1
 1

-.457221825409149
-.537482125493544
-.408725714134624
-2.23722937473523E-02

 .473890562404896
 .450586283818441
 .533146954290362
 .621910041349784

 8.53061157620403E-02
-2.00568701128889E-02
 6.12872810274759E-02
 .550084199523584

 .456499798142924
 .434570725847835
 .515861326936719
 .619382098553518

 .126651813806325
 .016057702432202
 9.68491956294829E-02
 .556410704914915

 .422417005683059
 .388577475261514
 .463709001755225
 .598336974774617

 .217555919057482
 .136321483771239
 .220209397197716
 .611153863564067

 .2151718890717
 .180546816907782
 .279678804822281
 .445369350499033

 .976546996211438
 .982575637573282
 .991328340559682
 .995945672004862

 .212648675506905
 .178973860321386
 .278466165148234
 .44446651378608

 1
 1
 1
 1

 2.52321356479528E-03
 1.57295658639578E-03
 1.21263967404692E-03
 9.02836712952359E-04

-1
-1
-1
-1

 9.81335983355373E-02
 9.35365783903573E-02
 4.63316547461741E-02
 1.71177840775767E-02

-1
-1
-1
-1

 9.81335983355373E-02
 9.35365783903573E-02
 4.63316547461741E-02
 1.71177840775767E-02

-1
-1
-1
-1

 7.63019781994211E-02
 8.10787622261406E-02
 9.63982396882344E-02
 8.92995792781583E-02

 .166666666668563
 3.11111111106562E-02
-.907780979827222
-.159235668789284

 9.78230489736169E-03
 9.00875135846607E-03
 7.36182522114947E-02
 4.55029703327989E-02

-1
-1
-1
-1

 .044509487283068
 4.18006063032363E-02
 4.4448756052148E-03
 3.75399505245995E-02

 1
 1
 1
 1

 2.05428402843862E-02
 2.70262540753982E-02
 1.58348693435884E-02
 5.68787129159986E-03

-1
-1
-1
-1

 4.89115244869075E-04
 2.16210032602414E-03
 0
 0

-1
-1
-1
-1

 0
 0
 1.66682835195697E-03
 0

 0
 0
-1
-1

 9.78230489736168E-04
 1.08105016301593E-03
 5.55609450652325E-04
 0

-1
-1
-1
-1

 0
 0
 2.77804725327112E-04
 5.68787129159986E-04

 0
 0
-1
-1

 1.19561504301185E-02
 1.04101126808794E-02
 3.88044695693808E-03
 1.93207056572062E-02

-1
-1
-1
-1

 1.19561504301185E-02
 1.04101126808794E-02
 3.88044695693808E-03
 1.93207056572062E-02

-1
-1
-1
-1

 6.49048166205783E-03
 6.30326602986239E-03
 2.12807238797739E-02
 1.78400534479386E-02

-1
-1
-1
-1

 3.83528461848882E-03
 4.78178802265479E-03
 1.30700508867955E-02
 1.71538975460948E-02

-1
-1
-1
-1

 1.77013136237854E-03
 4.34708002059315E-04
 4.69181313884643E-03
 3.43077950921896E-04

-1
-1
-1
-1

 8.85065681190464E-04
 8.6941600411863E-04
 3.51885985413196E-03
 0

-1
-1
-1
-1

 0
 2.17354001029657E-04
 0
 3.43077950921896E-04

 0
-1
-1
-1

 7.36001987515287E-03
 9.94108562604549E-03
 3.13522475726312E-03
 4.27944601939418E-03

-1
-1
-1
-1

 7.36001987515287E-03
 9.94108562604549E-03
 3.13522475726312E-03
 4.27944601939418E-03

-1
-1
-1
-1

 1.8400049687907E-03
 3.61494022765115E-03
 9.05731596539821E-03
 2.85296401292945E-03

-1
-1
-1
-1

 6.13334989597729E-04
 9.03735056912786E-04
 3.13522475726312E-03
 7.13241003232363E-04

-1
-1
-1
-1

 1.22666997919297E-03
 2.25933764228197E-03
 1.39343322544551E-03
 2.13972300969709E-03

-1
-1
-1
-1

 2.21266420297616E-03
 1.08677000514829E-03
 4.18911887398026E-04
 0

-1
-1
-1
-1

 2.21266420297616E-03
 0
 0
 0

-1
-1
-1
-1

 0
 1.08677000514829E-03
 4.18911887398026E-04
 0

 0
-1
-1
-1

 1.70802149001899E-03
 1.60155579705819E-03
 2.02841545476215E-03
 3.61134685180943E-04

-1
-1
-1
-1

 1.55274680910818E-03
 1.25836526911662E-03
 1.58745557329175E-03
 1.80567342590472E-04

-1
-1
-1
-1

 1.55274680910818E-04
 1.14396842647188E-04
 3.52767905176079E-04
 1.80567342590472E-04

-1
-1
-1
-1

 0
 2.28793685294376E-04
 8.81919762943213E-05
 0

 0
-1
-1
-1

 7.76373404552515E-04
 2.8599210661797E-04
 4.40959881470099E-04
 1.35425506942854E-03

-1
-1
-1
-1

 7.76373404552515E-04
 2.8599210661797E-04
 4.40959881470099E-04
 1.35425506942854E-03

-1
-1
-1
-1

 2.32912021366226E-04
 0
 9.26015751090373E-04
 5.41702027771415E-04

-1
-1
-1
-1

 2.32912021366226E-04
 0
 9.26015751090373E-04
 5.41702027771415E-04

-1
-1
-1
-1

 2.32912021366226E-04
 1.71595263970782E-04
 1.32287964441482E-04
 0

-1
-1
-1
-1

 2.32912021366226E-04
 1.71595263970782E-04
 1.32287964441482E-04
 0

-1
-1
-1
-1

 3.24524083103036E-02
 4.47920837385254E-02
 5.06089655963475E-02
 1.91491666817009E-02

-1
-1
-1
-1

 3.15207602248412E-02
 4.29617342561728E-02
 4.84659005724044E-02
 1.80657626261581E-02

-1
-1
-1
-1

 3.15207602248412E-02
 4.29617342561728E-02
 4.84659005724044E-02
 1.80657626261581E-02

-1
-1
-1
-1

 9.31648085462388E-04
 1.83034948235256E-03
 1.32287964440879E-03
 1.08340405554283E-03

-1
-1
-1
-1

 9.31648085462388E-04
 1.83034948235256E-03
 1.32287964440879E-03
 1.08340405554283E-03

-1
-1
-1
-1

 0
 0
 8.20185379534384E-04
 0

 0
 0
-1
-1

 1.63038414956138E-03
 1.20116684779548E-03
 1.5433595851464E-03
 1.89595709719995E-03

-1
-1
-1
-1

 1.63038414956138E-03
 1.20116684779548E-03
 1.5433595851464E-03
 1.89595709719995E-03

-1
-1
-1
-1

 1.63038414956138E-03
 1.20116684779548E-03
 1.5433595851464E-03
 1.89595709719995E-03

-1
-1
-1
-1

 1.08692276637352E-02
 9.20894583309436E-03
 1.32728924322721E-02
 0

-1
-1
-1
-1

 4.34769106549849E-03
 3.20311159411698E-03
 3.08671917029913E-03
 0

-1
-1
-1
-1

 4.34769106549849E-03
 3.20311159411698E-03
 6.17343834058139E-04
 0

-1
-1
-1
-1

 0
 0
 .002469375336241
 0

 0
 0
-1
-1

 .258873948013924
 .264222387462121
 .135304130029873
 .236777956338866

-1
-1
-1
-1

 .258873948013924
 .264222387462121
 .135304130029873
 .236777956338866

-1
-1
-1
-1

 .258873948013924
 .264222387462121
 .135304130029873
 .236777956338866

-1
-1
-1
-1

 .258873948013924
 .264222387462121
 .135304130029873
 .236777956338866

-1
-1
-1
-1

 .239200645942574
 .242120917462678
 .116677984636591
 .227244000650089

-1
-1
-1
-1

 1.41610508990233E-02
 1.62901103929635E-02
 1.39696090449626E-02
 8.08941694805313E-03

-1
-1
-1
-1

 3.02785627775434E-03
 3.43190527941564E-03
 2.11660743105467E-03
 0

-1
-1
-1
-1

 2.48439489457308E-03
 2.19641937882797E-03
 1.83439310691224E-03
 8.66723244434264E-04

-1
-1
-1
-1

 0
 1.83034948235501E-04
 7.05535810352641E-04
 5.7781549628951E-04

 0
-1
-1
-1

 .126230551846253
 .111468283475392
 .102267415710573
 4.42480273018079E-02

-1
-1
-1
-1

 3.10704636502358E-02
 5.20963221415689E-02
 4.78706047323634E-02
 9.55201242303595E-03

-1
-1
-1
-1

 3.24989907146038E-02
 3.54687410627913E-02
 4.78617855347763E-02
 4.02845741319663E-02

-1
-1
-1
-1

 3.24989907146038E-02
 3.54687410627913E-02
 4.78617855347763E-02
 4.02845741319663E-02

-1
-1
-1
-1

 3.24989907146038E-02
 3.54687410627913E-02
 4.78617855347763E-02
 4.02845741319663E-02

-1
-1
-1
-1

 3.24989907146038E-02
 3.53772235886735E-02
 4.75442944201179E-02
 4.02845741319663E-02

-1
-1
-1
-1

 .03163721623555
 3.43190527941871E-02
 4.57495877025284E-02
 .039950524548174

-1
-1
-1
-1

 8.61774479053873E-04
 1.05817079448649E-03
 1.79470671758944E-03
 3.34049583792373E-04

-1
-1
-1
-1

 0
 0
 1.7638395258804E-04
 0

 0
 0
-1
-1

 0
 0
 1.7638395258804E-04
 0

 0
 0
-1
-1

 0
 9.15174741177505E-05
 1.41107162070432E-04
 0

 0
-1
-1
-1

 3.58296326200995E-02
 4.56900989532634E-02
 8.25565090088114E-02
 1.99436629891176E-02

-1
-1
-1
-1

 1.92851153690865E-02
 2.52702625407792E-02
 4.24820749807928E-02
 1.21882956248568E-02

-1
-1
-1
-1

 1.92851153690865E-02
 2.52702625407792E-02
 4.24820749807928E-02
 1.21882956248568E-02

-1
-1
-1
-1

 8.54010745008238E-03
 .010581707944868
 3.41743908138988E-02
 7.22269370361887E-03

-1
-1
-1
-1

 1.00928542591795E-02
 1.37276211176748E-02
 7.93727786645877E-03
 4.96560192123797E-03

-1
-1
-1
-1

 4.65824042731194E-03
 6.29182634559841E-03
 7.71679792572296E-03
 1.80567342590472E-03

-1
-1
-1
-1

 5.04642712959056E-03
 5.43385002574756E-03
 0
 3.15992849533326E-03

-1
-1
-1
-1

 6.52153659824553E-04
 9.6093347823638E-04
 3.70406300435305E-04
 0

-1
-1
-1
-1

 4.34769106549408E-04
 8.00777898530317E-04
 0
 0

-1
-1
-1
-1

 0
 0
 3.70406300435305E-04
 0

 0
 0
-1
-1

 2.17384553275145E-04
 1.60155579706063E-04
 0
 0

-1
-1
-1
-1

 1.33924412285315E-02
 1.47057141222618E-02
 3.02851246593746E-02
 5.41702027771415E-03

-1
-1
-1
-1

 1.30741281326643E-02
 1.40021735399815E-02
 2.99235375565691E-02
 5.41702027771415E-03

-1
-1
-1
-1

 1.14903263873778E-02
 1.18972716352733E-02
 .028221432414096
 4.33361622217132E-03

-1
-1
-1
-1

 1.39747212819673E-03
 1.25836526911662E-03
 2.74277046274495E-02
 1.2639713981333E-03

-1
-1
-1
-1

 1.00928542591811E-02
 1.06389063661567E-02
 5.29151857764119E-04
 3.06964482403802E-03

-1
-1
-1
-1

 0
 0
 2.64575928882361E-04
 0

 0
 0
-1
-1

 9.31648085462388E-04
 1.14396842647188E-03
 1.14649569182015E-03
 1.08340405554283E-03

-1
-1
-1
-1

 9.31648085462388E-04
 1.14396842647188E-03
 1.14649569182015E-03
 1.08340405554283E-03

-1
-1
-1
-1

 6.52153659824112E-04
 9.6093347823638E-04
 5.55609450652958E-04
 0

-1
-1
-1
-1

 6.52153659824112E-04
 9.6093347823638E-04
 5.55609450652958E-04
 0

-1
-1
-1
-1

 3.18313095867176E-04
 7.03540582280207E-04
 3.61587102805481E-04
 0

-1
-1
-1
-1

 3.18313095867176E-04
 7.03540582280207E-04
 3.61587102805481E-04
 0

-1
-1
-1
-1

 3.18313095867176E-04
 7.03540582280207E-04
 3.61587102805481E-04
 0

-1
-1
-1
-1

 2.87258159684198E-03
 5.71412229022252E-03
 9.78930936864401E-03
 2.33834708654661E-03

-1
-1
-1
-1

 2.87258159684198E-03
 5.71412229022252E-03
 9.78930936864401E-03
 2.33834708654661E-03

-1
-1
-1
-1

 0
 1.48143911228335E-03
 3.91572374745671E-03
 3.34049583792373E-04

 0
-1
-1
-1

 0
 1.48143911228335E-03
 3.91572374745671E-03
 3.34049583792373E-04

 0
-1
-1
-1

 1.14903263873772E-03
 1.26980495337926E-03
 3.26310312287873E-04
 6.68099167584745E-04

-1
-1
-1
-1

 1.14903263873772E-03
 1.26980495337926E-03
 3.26310312287873E-04
 6.68099167584745E-04

-1
-1
-1
-1

 2.87258159685012E-04
 2.11634158897298E-04
 6.52620624575747E-04
 0

-1
-1
-1
-1

 2.87258159685012E-04
 2.11634158897298E-04
 6.52620624575747E-04
 0

-1
-1
-1
-1

 2.79494425639472E-04
 0
 0
 0

-1
-1
-1
-1

 2.79494425639472E-04
 0
 0
 0

-1
-1
-1
-1

 2.79494425639472E-04
 0
 0
 0

-1
-1
-1
-1

 2.79494425639472E-04
 0
 0
 0

-1
-1
-1
-1

 4.16058507499881E-02
 4.04678830864231E-02
 5.09926006932405E-02
 2.72837254654216E-02

-.31591714872186
-.481837455830608
-.500864752680632
-.500992720054192

 2.30349989130801E-02
 1.91786306697909E-02
 2.63561721154925E-02
 1.76233726368313E-02

 .235591506572332
 9.33492394866101E-02
-3.42981428810262E-02
-.227459016395393

 1.54731219527363E-02
 1.12280501058114E-02
 1.28142941555332E-02
 8.52277857025358E-03

 .839438033115955
 .867549668874161
 .986235375086042
 .597457627117856

 1.42309245054523E-02
 1.04844706286052E-02
 1.27261021792392E-02
 6.8073888156441E-03

 1
 1
 1
 1

 1.42309245054523E-02
 1.04844706286052E-02
 1.27261021792392E-02
 6.8073888156441E-03

 1
 1
 1
 1

 1.24219744728402E-03
 7.43579477206111E-04
 8.81919762940198E-05
 1.71538975460948E-03

-1
-1
-1
-1

 1.00928542591811E-03
 7.43579477206111E-04
 0
 1.44453874072377E-03

-1
-1
-1
-1

 2.32912021365912E-04
 0
 8.81919762940198E-05
 2.70851013885708E-04

-1
-1
-1
-1

 6.3041520449695E-03
 6.40622318824254E-03
 1.24703454479833E-02
 9.10059406657775E-03

-1
-1
-1
-1

 6.3041520449695E-03
 6.40622318824254E-03
 1.24703454479833E-02
 9.10059406657775E-03

-1
-1
-1
-1

 6.3041520449695E-03
 6.40622318824254E-03
 1.24703454479833E-02
 9.10059406657775E-03

-1
-1
-1
-1

 1.25772491537422E-03
 1.54435737573704E-03
 .001071532511976
 0

-1
-1
-1
-1

 1.25772491537422E-03
 1.54435737573704E-03
 .001071532511976
 0

-1
-1
-1
-1

 1.25772491537422E-03
 1.54435737573704E-03
 .001071532511976
 0

-1
-1
-1
-1

 1.38427378031794E-02
 1.33729909054571E-02
 1.37844058947693E-02
 4.7850345786475E-03

-1
-1
-1
-1

 9.09133256731182E-03
 9.25470457015838E-03
 8.54580250289751E-03
 2.18486484534471E-03

-1
-1
-1
-1

 9.09133256731182E-03
 9.25470457015838E-03
 8.54580250289751E-03
 2.18486484534471E-03

-1
-1
-1
-1

 7.45318468370414E-03
 6.04015329177447E-03
 5.92650080696392E-03
 4.33361622217132E-04

-1
-1
-1
-1

 1.18785130896722E-03
 1.55579705999968E-03
 1.72415313654886E-03
 1.22785792961521E-03

-1
-1
-1
-1

 4.50296574640458E-04
 1.65875421838423E-03
 6.3939182813208E-04
 0

-1
-1
-1
-1

 0
 0
 2.55756731252657E-04
 5.23645293512368E-04

 0
 0
-1
-1

 4.75140523586762E-03
 4.11828633529877E-03
 5.23860339187183E-03
 2.60016973330279E-03

-1
-1
-1
-1

 4.75140523586762E-03
 4.11828633529877E-03
 5.23860339187183E-03
 2.60016973330279E-03

-1
-1
-1
-1

 1.9564609794729E-03
 2.05914316764939E-03
 3.9686389332336E-03
 1.62510608331425E-03

-1
-1
-1
-1

 2.79494425639472E-03
 2.05914316764939E-03
 1.26996445863823E-03
 9.75063649988547E-04

-1
-1
-1
-1

 2.14279059656928E-03
 5.27369444603244E-03
 7.17000767270411E-03
 4.87531824994274E-03

-1
-1
-1
-1

 3.72659234185962E-04
 2.64256706514711E-03
 3.09553836791621E-03
 2.8710207471885E-03

-1
-1
-1
-1

 3.72659234185962E-04
 2.19641937882307E-03
 2.75158966036835E-03
 2.16680811108566E-03

-1
-1
-1
-1

 3.72659234185962E-04
 2.19641937882307E-03
 2.75158966036835E-03
 2.16680811108566E-03

-1
-1
-1
-1

 0
 4.46147686324034E-04
 3.43948707547853E-04
 7.0421263610284E-04

 0
-1
-1
-1

 0
 4.46147686324034E-04
 3.43948707547853E-04
 7.0421263610284E-04

 0
-1
-1
-1

 .001234433713241
 5.89143739633019E-04
 1.19500127878636E-03
 9.29921814340929E-04

-1
-1
-1
-1

 .001234433713241
 5.89143739633019E-04
 1.19500127878636E-03
 9.29921814340929E-04

-1
-1
-1
-1

 7.9966460669071E-04
 5.89143739633019E-04
 4.54188677915755E-04
 9.29921814340929E-04

-1
-1
-1
-1

 4.34769106550289E-04
 0
 7.4081260087061E-04
 0

-1
-1
-1
-1

 0
 8.92295372648068E-04
 6.87897415093354E-04
 0

 0
-1
-1
-1

 0
 8.92295372648068E-04
 6.87897415093354E-04
 0

 0
-1
-1
-1

 0
 8.92295372648068E-04
 6.87897415093354E-04
 0

 0
-1
-1
-1

 3.64895500140421E-04
 8.06497740662677E-04
 4.14502288581893E-04
 0

-1
-1
-1
-1

 3.64895500140421E-04
 8.06497740662677E-04
 4.14502288581893E-04
 0

-1
-1
-1
-1

 3.64895500140421E-04
 8.06497740662677E-04
 4.14502288581893E-04
 0

-1
-1
-1
-1

 0
 0
 7.93727786647083E-04
 5.41702027771415E-04

 0
 0
-1
-1

 0
 3.43190527941564E-04
 5.29151857764119E-04
 0

 0
-1
-1
-1

 0
 3.43190527941564E-04
 5.29151857764119E-04
 0

 0
-1
-1
-1

 0
 3.43190527941564E-04
 5.29151857764119E-04
 0

 0
-1
-1
-1

 1.70802149001899E-04
 0
 4.54188677915091E-04
 5.32673660641892E-04

-1
-1
-1
-1

 1.70802149001899E-04
 0
 4.54188677915091E-04
 5.32673660641892E-04

-1
-1
-1
-1

 0
 0
 1.63155156144494E-04
 3.34049583792373E-04

 0
 0
-1
-1

 1.70802149001899E-04
 0
 2.91033521770597E-04
 1.98624076849519E-04

-1
-1
-1
-1

 4.65824042731509E-04
 1.02957158382102E-03
 2.11660743105467E-03
 0

-1
-1
-1
-1

 4.65824042731509E-04
 1.02957158382102E-03
 2.11660743105467E-03
 0

-1
-1
-1
-1

 4.65824042731509E-04
 1.02957158382102E-03
 2.11660743105467E-03
 0

-1
-1
-1
-1

 4.65824042731509E-04
 1.02957158382102E-03
 2.11660743105467E-03
 0

-1
-1
-1
-1

 2.11949939442785E-03
 1.61299548132156E-03
 1.56540757921992E-03
 0

-1
-1
-1
-1

 1.4440545324667E-03
 1.06389063661505E-03
 5.46790253022923E-04
 0

-1
-1
-1
-1

 1.4440545324667E-03
 1.06389063661505E-03
 5.46790253022923E-04
 0

-1
-1
-1
-1

 1.4440545324667E-03
 1.06389063661505E-03
 5.46790253022923E-04
 0

-1
-1
-1
-1

 0
 0
 5.55609450652958E-04
 0

 0
 0
-1
-1

 0
 0
 5.55609450652958E-04
 0

 0
 0
-1
-1

 0
 0
 5.55609450652958E-04
 0

 0
 0
-1
-1

 6.75444861961144E-04
 5.49104844706503E-04
 4.63007875544041E-04
 0

-1
-1
-1
-1

 4.42532840595232E-04
 4.34708002059315E-04
 4.1891188739688E-04
 0

-1
-1
-1
-1

 4.42532840595232E-04
 4.34708002059315E-04
 4.1891188739688E-04
 0

-1
-1
-1
-1

 2.32912021365912E-04
 1.14396842647188E-04
 4.40959881471606E-05
 0

-1
-1
-1
-1

 2.32912021365912E-04
 1.14396842647188E-04
 4.40959881471606E-05
 0

-1
-1
-1
-1
